# Machine Learning Exciton Hamiltonians in Light-Harvesting Complexes

**DOI:** 10.1021/acs.jctc.2c01044

**Published:** 2023-01-26

**Authors:** Edoardo Cignoni, Lorenzo Cupellini, Benedetta Mennucci

**Affiliations:** Dipartimento di Chimica e Chimica Industriale, University of Pisa, via G. Moruzzi 13, 56124Pisa, Italy

## Abstract

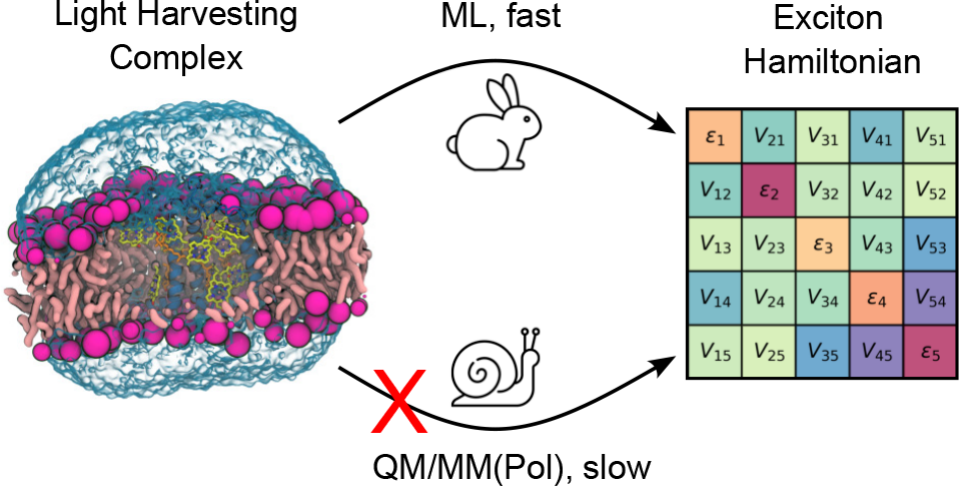

We propose a machine learning (ML)-based strategy for an inexpensive calculation of excitonic properties of light-harvesting complexes (LHCs). The strategy uses classical molecular dynamics simulations of LHCs in their natural environment in combination with ML prediction of the excitonic Hamiltonian of the embedded aggregate of pigments. The proposed ML model can reproduce the effects of geometrical fluctuations together with those due to electrostatic and polarization interactions between the pigments and the protein. The training is performed on the chlorophylls of the major LHC of plants, but we demonstrate that the model is able to extrapolate well beyond the initial training set. Moreover, the accuracy in predicting the effects of the environment is tested on the simulation of the small changes observed in the absorption spectra of the wild-type and a mutant of a minor LHC.

## 1 Introduction

Photosynthetic light harvesting is made possible by aggregates of pigments embedded in a protein matrix, the light-harvesting complexes (LHCs). The pigments in LHCs are responsible for both absorbing sunlight and funneling the resulting excitation energy toward the reaction centers.^1−3^ Their photophysics is the result of the interactions between the pigments and the interactions between each pigment with the embedding protein. The interaction with the protein matrix shapes the individual energies of each pigment, also called site energies, whereas the closely spaced arrangement of pigments enables excitonic coupling between them. These two parameters tune the optical properties of LHCs, resulting in rich and complex spectra of the multichromophoric aggregate as compared to the single chromophore.^4−7^ In addition, they determine the regime and direction of excitation energy transfer (EET) within individual LHCs and among different LHCs in the photosynthetic machinery.^8,9^

Modeling LHCs is extremely challenging, as they combine the complexity of proteins with the intrinsic quantum nature of the light response of the multichromophoric aggregate.^10^ A very effective strategy to get through these difficulties is to use classical molecular dynamics (MD) simulations to generate conformational ensembles of LHCs at the desired external conditions, in combination with hybrid quantum mechanics (QM)-classical descriptions of the embedded aggregate. In particular, methods coupling atomistic molecular mechanics (MM) to QM descriptions (QM/MM) have been shown to be successful in describing LHCs.^7,11−15^ Within QM/MM, the environment interacts with the QM subsystem through electrostatic interactions of a classical nature. In its standard formulation, known as electrostatic embedding QM/MM (EE-QM/MM), each MM atom is assigned a fixed charge (i.e., the charge it has in a classical MM force-field (FF)), and the corresponding set of MM point charges interacts with the electrostatic potential of the QM part. This results in a polarization of the QM system, but it completely discards the polarization of the MM part due to the presence of the QM molecule. This contribution can be recovered by making the MM environment polarizable, in what is known as polarizable embedding QM/MM (QM/MMPol). Here, the mutual polarization between the QM and MM subsystems is included, which plays a key role in the description of biological matrices.^16^

When modeling LHC, QM/MM(Pol) calculations should be run for many configurations along the dynamics in order to recover the distributions of site energies and couplings with reasonable statistical uncertainty.^7^ While the above strategy is known to work well for a variety of systems, its fundamental limitation is the computational cost. In recent years, several authors have tried to bypass the computational cost of expensive QM calculations by exploiting machine learning (ML) techniques. Some works focused on obtaining estimates of excitation energies and couplings in vacuum.^17−20^ Inclusion of the environment effects poses further challenges, and several works have tried to include these effects, either in excited-state properties^21,22^ or by developing ground-state QM/MM potentials.^23−29^

In a previous work^30^ we have presented a ML approach to estimate excitonic couplings in LHCs with an accuracy comparable to that of the reference time-dependent density functional theory (TD-DFT) calculations while being orders of magnitude faster. In this work we develop a model for estimating site energies, thus providing a ML estimate for the full exciton Hamiltonian. The ML model employed is Gaussian Process Regression (GPR).^31^ GPR is a powerful nonlinear regression algorithm widely employed in the literature.^32^ Although more complex models like neural networks (NNs) are known to scale better with large amounts of training data, GPR models generally perform as well as NNs on small datasets, with the advantage of being more transparent to the user. Furthermore, by manipulating the kernel of a GPR model it is possible to build physical constraints directly inside the model, facilitating the learning process considerably. Rather than building a single model for predicting the excitation energies of the embedded pigments, we exploit the freedom in composing the GPR kernel to develop a sequential strategy. Namely, we first model the excitation energies *in vacuo* and then add on top of those the corrections for the electrostatic and polarization effects due to the environment.

As an example, we consider the aggregate of chlorophylls (Chls) present in various LHCs: the major light-harvesting complex II (LHCII) of plants, the minor antenna CP29, and the light-harvesting complex stress-related 1 (LHCSR1) of mosses. We train our ML model on Chl *a* and Chl *b* pigments embedded in LHCII and show its performance for CP29 and LHCSR1. We further showcase two example applications for the analysis of LHCs: the estimation of the influence of protein residues on the excitation energy of Chls, and the calculation of the absorption spectrum of CP29 and one of its mutants (CP29-H111N). The ML models presented here and in ref (30) are implemented in a Python package, excipy, available for download under the LPGL license agreement^33^ (https://github.com/Molecolab-Pisa/excipy).

## 2 Methods

The excited states of multichromophoric systems can be described within the (Frenkel) exciton model. In the exciton model, the excited states of the system are represented as linear combinations of excited states localized on each chromophore. Assuming for simplicity that each chromophore contributes with one excitation, the resulting Hamiltonian reads
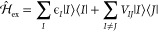
1where ϵ_*I*_ is the excitation energy of the state localized on chromophore *I*, also called site energy, and *V*_*IJ*_ is the electronic coupling between the transitions of pigments *I* and *J*. Site energies can be obtained from QM/MM(Pol) calculations on each single chromophore, whereas the electronic couplings can be computed from the transition density of each excitation.^34^

Clearly, both the site energies ϵ_*I*_ and the couplings *V*_*IJ*_ depend not only on the geometry of the chromophores but also on the position of the environment atoms. We have previously developed a ML approach for estimating electronic couplings,^30^ which we now combine with a ML model for excitation energies of the single chromophores.

### 2.1 Machine Learning Models of Excitation Energies

To obtain the excitation energy of a given state, ϵ, we build a surrogate model,
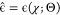
2that provides an estimate ϵ̂ of the excitation energy given a suitable, mathematical encoding χ of the chromophore (and possibly the environment) geometry, and a set of additional parameters Θ.

**Figure 1:**
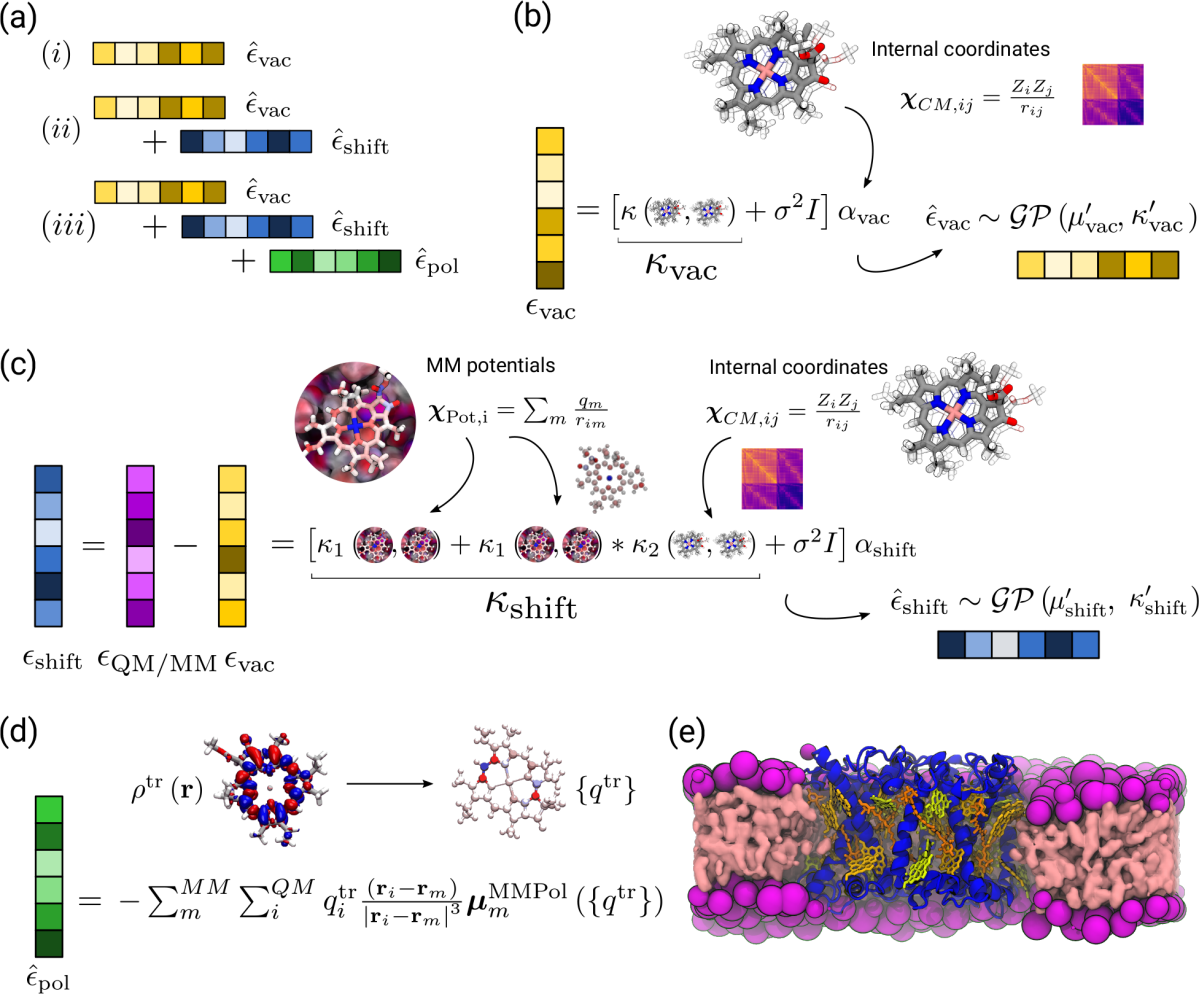
Overview of the ML models developed in this work. (a) Summary of predictions of the ML models: (i) vacuum, (ii) electrostatic embedding (EE), and (iii) polarizable embedding. (b) Vacuum ML model. The internal geometry of the pigments is encoded as a Coulomb Matrix and a nonlinear kernel κ_vac_. A Gaussian Process Regression (GPR) model is fit to reproduce the vacuum excitation energies ϵ_vac_ (yellow squares), solving for α_vac_. Predictions ϵ̂_vac_ are drawn from a Gaussian process (GP) with posterior mean μ_vac_^′^ and covariance κ_vac_^′^. (c) Electrostatic embedding ML model. The internal geometry of the pigments is encoded analogously to the vacuum case. MM electrostatic potentials are used as additional features with a linear kernel. The linear and nonlinear kernels are combined into the resulting κ_shift_ kernel. A GPR model is fit to reproduce the electrochromic shift ϵ_shift_ = ϵ_QM/MM_ – ϵ_vac_ (blue squares), solving for α_shift_. Predictions ϵ̂_shift_ are drawn from a GP with posterior mean μ_shift_^′^ and covariance κ_shift_^′^. (d) Polarizable embedding ML model. The additional contribution is estimated via a TrEsp representation of the QM charge distribution ρ_tr_, where TrEsp charges are estimated through a linear model as explained in ref (30). (e) A representation of the system used to construct the training dataset. LHCII protein is represented in blue, chlorophylls *a* and *b* are represented in yellow, carotenoids are represented in orange, and membrane is represented in pink.

Here, the strategy is to split the problem in parts, by first modeling the QM excitation energies *in vacuo* and then adding corrections for the environment, including both electrostatic and polarization effects. Within this framework, eq 2 can be rewritten as
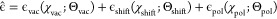
3where ϵ_vac_(χ_vac_; Θ_vac_) represents the vacuum model, ϵ_shift_(χ_shift_; Θ_shift_) represents the environment shift needed to recover an electrostatic embedding, and ϵ_pol_(χ_pol_; Θ_pol_) is the polarization term (see Figure 1a). This step-wise separation allows us to independently control each model and easily impose physical constraints.

In this work we model ϵ_vac_(χ_vac_; Θ_vac_) and ϵ_shift_(χ_shift_; Θ_shift_) as a Gaussian process (GP) in what is known as standard Gaussian Process Regression (GPR).^31,32,35−37^ As detailed in the following, we will instead use an analytical expression for ϵ_pol_(χ_pol_; Θ_pol_).

A GPR model defines a prior distribution for a target ϵ as a GP, ϵ(χ) ≈ , which is fully specified by its prior mean μ(χ) =  and covariance (also known as kernel) κ(χ,χ′) =  functions, where χ denotes an input vector,  denotes an expectation, and we have omitted the dependence on the hyperparameters Θ for simplicity. Collecting the training inputs into a vector **χ** = (χ_1_, χ_2_, ..., χ_*N*_) and the corresponding mean-free targets into **ϵ** = (ϵ_1_ – μ(χ_1_), ϵ_2_ – μ(χ_2_), ..., ϵ_*N*_ – μ(χ_*N*_)), we take the prediction ϵ̅(χ_*_) for a new point χ_*_ as the posterior mean μ′(χ_*_):
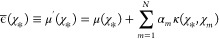
4where the expansion coefficients α_*m*_ are determined from the resolution of the following linear system:
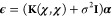
5where **K**(**χ**, **χ**)_*ij*_ = κ(χ_*i*_, χ_*j*_) is a matrix of kernel evaluations over the training inputs, σ^2^ is a hyperparameter that models the noise associated with each observation, and **I** is an *N*×*N* identity matrix. The variance of each prediction, var(ϵ̂), can be defined as the diagonal element of the posterior covariance, κ′(χ_*_, χ_*_):
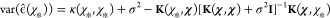
6where **K**(χ_*_, **χ**)_*i*_ = κ(χ_*_, χ_*i*_) is a vector of kernel evaluations of the new point and the training inputs.

Additional hyperparameters **Θ** usually enter the prior mean μ(·) and covariance κ(·, ·) functions and can be set by maximizing the log marginal likelihood.^31,32^ The power of GP regression stems mainly from the freedom of choosing the prior kernel. In fact, as any symmetric and positive semidefinite function is a valid covariance function, one can in principle incorporate physical requirements inside the kernel, considerably improving the learning efficiency. Moreover, several mathematical operations between kernels yield a new kernel as a result,^31^ making GPR a very flexible and powerful algorithm. A limitation of GPR modeling is that its memory requirement scales as *O*(*N*^2^), while the computational cost scales as *O*(*N*^3^), where *N* is the number of training points. Several types of sparse GPR methods have been developed to mitigate this problem.^32^ In the present case, however, the limited number of training points allowed us to use the full GPR algorithm.

#### 2.1.1 Vacuum ML Model

Vacuum site energies ϵ_vac_ are modeled with a GPR model (see Figure 1a and b), taking as input the chromophore geometry encoded as a Coulomb matrix^38^ (CM), ϵ̂_vac_ = ϵ_vac_(χ_CM_), where
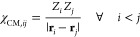
7where *Z*_*i*_ is the atomic number of the *i*th atom, and *r*_*ij*_ is the distance between atoms *i* and *j*. The diagonal part of the CM, commonly written as 0.5*Z*_*i*_^2.4^, is here ignored, as in our case it is constant (and therefore uninformative). For our purpose, we have found it beneficial to exclude hydrogen atoms from the CM: this helps reduce the risk of overfitting, leaving less room for the regression algorithm to learn by heart the training data. Furthermore, it removes identical atoms, which can be beneficial when dealing with descriptors such as the CM which are not permutation invariant.^39^ In fact, identical atoms must be handled with care when a CM encoding is employed.^30,39^

We note that, as the CM uses inverse distances between all the atoms, it can describe changes in bond distances, angles, and torsions, as well as capturing more complex relations between distant atoms. Although other *ad-hoc* descriptors could be devised for each regression problem, the CM has the advantage of being totally general, and therefore applicable on molecules different from the ones used in this work. It has been shown empirically that the CM, being a global descriptor, works well for modeling excitation properties.^17−19,21^ In our previous work, we have employed it with Ridge regression to learn transition charges associated with Chls embedded in LHCs.^30^

For our present task of learning excitation energies, we have found it essential to introduce nonlinearity in the regression algorithm. The nonlinearity has been introduced with a Matern kernel, and the prior mean is defined as the average of the training energies:
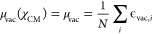
8
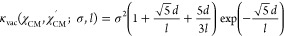
9where ϵ_vac,*i*_ is the vacuum excitation energy of the *i*th training target, *d* = |χ_CM_ – χ_CM_^′^| is the euclidean distance between χ_CM_ and χ_CM_^′^, and σ and *l* are two kernel hyperparameters. In this work, they have been determined via maximization of the log marginal likelihood. The vacuum ML model is represented schematically in Figure 1b.

We finally note that the descriptor and regression algorithm employed here were chosen as the best-performing ones in several tests with different models and descriptors (details are reported in Table S1 of the Supporting Information).

#### 2.1.2 Electrostatic Embedding ML Model

In order to predict the effects on the site energies due to an electrostatic embedding (EE), we define the electrochromic shift ϵ_shift_ = ϵ_QM/MM_ – ϵ_vac_, where ϵ_QM/MM_ and ϵ_vac_ are evaluated at the same geometry, with and without the MM charges. We build a GPR model to estimate ϵ_shift_, which is then added to the vacuum one to recover the full site energy in the environment: ϵ̂_QM/MM_ = ϵ̂_vac_ + ϵ̂_shift_ (see Figure 1a and c).

The presence of an atomistic and heterogeneous environment polarizing the QM density requires a specific featurization and a more complex kernel for estimating ϵ_shift_. As the size of the system grows considerably compared to the vacuum case, we seek features whose number does not depend on the number of environment atoms. Furthermore, the interaction between QM and MM subsystems is remarkably more complex than in the vacuum case, where only the description of the internal geometry of the pigment was needed.

In order to define a suitable kernel and featurization, we observe that (i) given a QM subsystem with a fixed geometry, the EE-QM/MM interaction is an electrostatic interaction between the QM density ρ(**r**) and the MM point charges **q**,
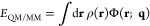
where Φ(**r**; **q**) is the MM electrostatic potential at point **r**; (ii) given a fixed arrangement of MM charges **q**, the QM response will be different for different QM geometries, i.e., the QM response has a dependence on the QM internal geometry.

Therefore, a natural encoding of the environment is the electrostatic potential due to the MM point charges on the QM atoms:
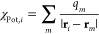
10where *m* runs over the MM atoms, *i* refers to the *i*th QM atom, and *q*_*m*_ is the atomic charge of atom *m*. We note that this encoding is extremely memory efficient, as χ_Pot_ is a vector of length *n*, the number of QM atoms. Furthermore, this featurization does not depend on the choice of the target molecule, which makes it applicable in arbitrary general settings. The potential in eq 10 can be computed including MM atoms up to a certain distance threshold with the QM region, in order to reduce the cost of computing χ_Pot_. In this work, we include MM residues within 30 Å of the QM subsystem. To characterize the internal geometry of the QM system we use the same CM descriptor as in the vacuum case, eq 7.

We then define the prior GP with the following mean and composite kernel:
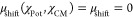
11
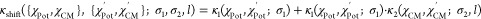
12
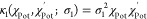
13
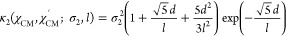
14where σ_1_, σ_2_, and *l* are kernel hyperparameters. The κ_1_ term in eq 12 is a linear kernel operating on the MM electrostatic potential, and represents the direct interaction between the QM and MM regions. It is mathematically equivalent to the expression ⟨Φ(**C**), **q**⟩ = ∑_*im*_ *c*_*i*_*q*_*m*_*r*_*im*_^–1^, where Φ(**C**) is the electrostatic potential generated by effective QM charges **C** = (*c*_1_, *c*_2_, ..., *c*_*n*_), determined as the regression coefficients in ordinary linear regression (OLS). The second term is a nonlinear response of the QM internal degrees of freedom, weighted by the magnitude of the interaction energy between the QM and MM parts. The zero mean defined in eq 11 ensures that, for zero MM potentials acting on the QM system (vacuum case), the electrostatic shift is predicted to be exactly zero. As in the vacuum case, kernel hyperparameters are determined by maximization of the log marginal likelihood. The EE ML model is represented schematically in Figure 1c.

#### 2.1.3 Polarizable Embedding ML Model

A polarizable environment introduces an additional term in the excitation energy which is not present in EE-QM/MM.^16,40^ This term can be interpreted as the resonant response of the MM polarizable sites to the transition density associated with the electronic excitation. For this reason, it has been classified as a dispersion-like or resonance contribution.^41^

Within an induced-dipole formulation of polarizable embedding, this contribution can be written as^16,40^
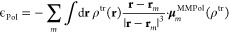
15where *m* runs over the polarizable MM sites, and **μ**_*m*_^MMPol^(ρ^tr^) is the induced dipole on MM atom *m* due to the transition density ρ^tr^. As we have shown in our previous work,^30^ we can approximate the transition density ρ^tr^ as a set of transition charges {*q*^tr^} and estimate the polarization contribution as
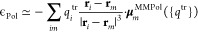
16where *i* runs over the QM atoms, and the induced dipoles are now dependent on the set of transition charges. This expression opens up the possibility of a fast computation of the polarization contribution, as it is possible to estimate transition charges with a Ridge regression model efficiently. In this work, the transition charges in the environment are obtained from a linear model, as described in ref (30) (see also below), and used to compute the polarization term. The polarizable ML model is represented schematically in Figure 1d.

### 2.2 Machine Learning Model for Couplings

We briefly summarize here the approach used to estimate electronic couplings. When considering bright transitions, the electronic coupling *V*_*IJ*_ can be accurately described as the Coulomb interaction between the transition densities associated with chromophores *I* and *J*. By projecting each transition density onto a set of atomic charges {*q*^tr^}, the Coulomb coupling term can be obtained as
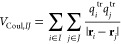
17where *i* and *j* are indices of QM atoms in chromophores *I* and *J*, respectively. This approach is called transition charges from electrostatic potentials (TrEsp),^42^ as these charges are obtained from a fit of the electrostatic potential generated by the transition density.

The bare Coulomb coupling (eq 17) is indirectly affected by the environment through the change of transition charges going from the isolated to the embedded pigment. However, the environment also directly affects the coupling through a screening of the Coulomb interaction. This explicit effect can only be taken into account if the environment model is polarizable.^10,34^ Using the same TrEsp representation of the QM transition charges used for the polarizable model, this screening term can be expressed as^30^
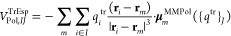
18This equation is similar to eq 16, but here **μ**_*m*_^MMPol^({*q*^tr^}_*J*_) are the dipoles induced by the transition density of chromophore *J* and interact by the field generated by the transition charges of chromophore *I*.

#### 2.2.1 Estimation of Transition Charges

To estimate transition charges, we use the linear model devised in ref (30). Briefly, transition charges *in vacuo* are estimated with a Ridge regression linear model, using as input the CM encoding (eq 7). The effect of the environment on the transition charges is modeled as a scaling of the transition charges by a factor γ. This factor is estimated separately for Chl *a* and Chl *b* through a Bayesian linear model.

We use the model as trained in ref (30) to estimate the transition charges that are used in eqs 17 and 18 for computing electronic couplings, as well as in eq 16 to compute the polarization contribution to the excitation energy.

## 3 Computational Details

### 3.1 Excitation Energy Calculations

All excitation energies were calculated at the TD-DFT M062X/6-31G(d) level of theory. This level of theory was chosen as it has been previously used in our group to successfully model LHCs.^7,43,44^ Furthermore, it yields well-separated Q_*y*_ and Q_*x*_ states, i.e., a well-defined regression target. QM/MM calculations included all MM atoms (protein, membrane, water, and ions) up to 30 Å from the QM region. In all calculations, the phytyl Chl tail was excluded from the QM part, cutting it after the first aliphatic carbon. EE-QM/MM charges were taken from the AMBER ff99SB^45^ force field, while for QM/MMPol calculations we used the AMBER AL polarizabilities^46^ and fixed charges consistent with polarization. All calculations are performed with Gaussian 16^47^ or a locally modified version for QM/MMPol calculations.

### 3.2 Generation of the Training Dataset

The training dataset was generated similarly to that described in ref (30). Chlorophyll geometries have been extracted from a classical MD simulation of LHCII embedded in a 1,2-dioleoyl-*sn*-glycero-3-phosphocoline (DOPC) membrane employed in several works by some of us.^48,49^ 240 frames separated by at least 10 ns from each other have been selected, for a total of 5760 training samples for Chl *a* and 4320 training samples for Chl *b*. The training targets are the Q_*y*_ excitations of Chls *a* and *b*, calculated at the QM or EE-QM/MM levels as described above. The training dataset and Python scripts to train the models are provided in a Zenodo repository.^33^

### 3.3 Generation of the Test Datasets

Chlorophyll geometries for CP29 and LHCSR1 LHCs analyzed in Section 4.1 were extracted from classical MD simulations previously analyzed by some of us.^44,50^ For CP29, we have extracted 100 frames, for a total of 1300 test samples, while for LHCSR1 we have extracted 408 frames, for a total of 3264 test samples. Excitation energies in vacuum were calculated as described above.

The scan over the improper dihedral of Chl *a* analyzed in Section 4.1 is described in the Supporting Information.

The performance of the EE-QM/MM ML model (Section 4.2) is tested on some Chls present in CP29 (*a*609, *a*612, *a*616, *b*606). For each Chl, 50 geometries were extracted from the classical MD of CP29,^50^ by first computing the MM electrostatic potential on the Chl atoms and then using farthest point sampling (FPS)^51^ to adequately sample the range of potentials felt by the Chl. EE-QM/MM excitation energies were obtained as explained above.

Chlorophyll geometries in methanol (Section 4.2) were extracted from a classical MD simulation, the details of which are reported in the Supporting Information. A total of 100 Chl *a* geometries were extracted analogously to those in CP29, i.e., by first computing MM potentials and then selecting structures with FPS. EE-QM/MM excitation energies were obtained as explained above.

Finally, for the analysis of the QM/MMPol ML model (Section 4.3), we have extracted geometries for Chls *a*603 and *a*609 from the classical MD trajectories of CP29.^50^ For each Chl, we have extracted 100 frames and computed the QM/MMPol excitation energies as described above.

### 3.4 Machine Learning Scores

In order to test the performance of the ML models, we have employed two scores. The first is the mean absolute error (MAE), defined as
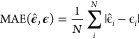
19where **ϵ̂** and **ϵ** denote the predicted and the target energies, and the sum runs over the *N* predictions. The second is the squared Pearson correlation coefficient (*r*-squared), defined as
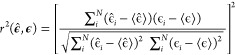
20

## 4 Results and Discussion

### 4.1 Vacuum ML Model

**Figure 2:**
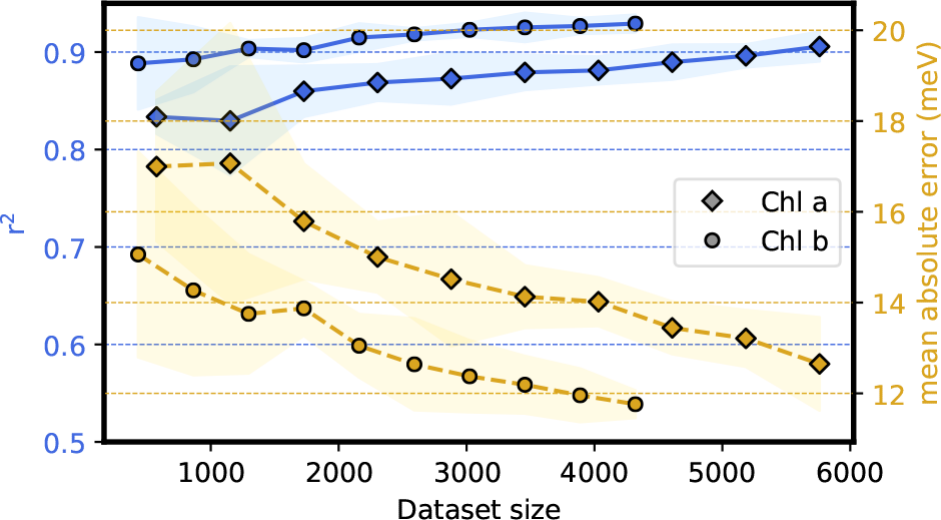
Learning curves for vacuum site energies ϵ̂_vac_ of chlorophylls in LHCII. Blue lines report Pearson’s *r*-squared, and yellow lines report the mean absolute error (MAE), both evaluated on the validation test with 5-fold cross-validation (CV-5). The uncertainty is computed as twice the standard deviation of the validation score and shown as a shaded region around the corresponding curve. The horizontal axis reports the dataset size used to perform CV-5. Diamond markers correspond to Chl *a*, while circles correspond to Chl *b*.

We first test the performance of our vacuum ML model in predicting the site energies of Chls *a* and *b*. Figure 2 shows the learning curves obtained with 5-fold cross-validation (CV-5) for the vacuum ML model, where both the Pearson’s *r*-squared and the mean absolute error (MAE) have been computed on the validation folds. Points correspond to the mean score, and the shaded region represents the uncertainty, computed as twice the standard deviation of the validation scores. For both Chl *a* and *b*, we observe a consistent decrease of the MAE and increase of *r*^2^ as the training set increases.

Both scores do not reach a clear plateau for our maximum train set size, indicating that it is possible to slightly improve the prediction error with even more QM calculations. Our prediction error (∼12.6 meV for Chl *a*, ∼11.8 meV for Chl *b*) compares well with what obtained by Häse et al. for BChls in the Fenna–Matthews–Olson (FMO) complex for a comparable training set size.^21^ The reduction of the validation error with increasing training set size is an indication of the robustness of the model’s prediction error. Interestingly, we also find that learning the site energy of Chl *b* is slightly easier than learning that of Chl *a*, due to the reduced conformational freedom of Chl *b* as compared to Chl *a* in LHCII (see Figure S1).

**Figure 3:**
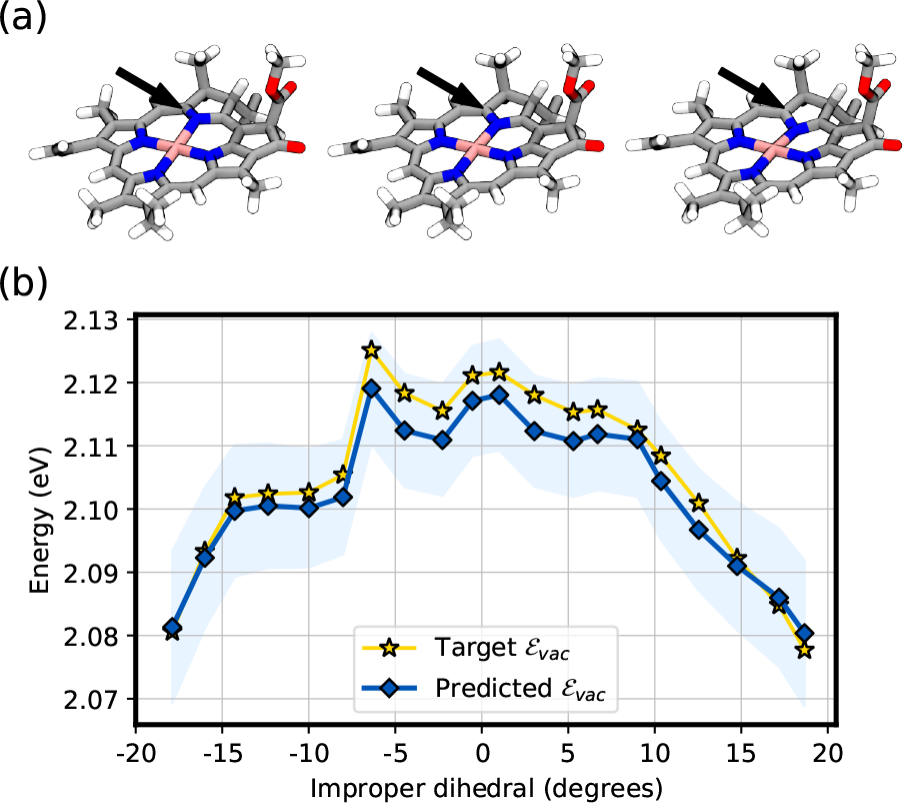
Vacuum ML model predictions along a scan over an improper dihedral of Chl *a*. (a) Illustration of the scan. The black arrow indicates the nitrogen atom that is pushed through the Chl’s porphyrin ring. (b) Vacuum excitation energy predicted by the vacuum ML model (blue line with circle markers) and target excitation energy computed with TD-DFT (yellow line with star markers). The uncertainty (shaded blue region) is computed as twice the square root of the posterior variance matrix eq 6.

In order to test the model against out of sample geometries, we have performed a relaxed scan over the improper dihedral formed by atoms NA-C1-MG-C4 in Chl *a* (see Figure 3a and Figure S2a). More details on how the scan is performed are provided in the Supporting Information. Along the scan, the nitrogen atom (NA) moves from one side of the Chl plane to the other, considerably impacting the planarity of the ring. This is reflected in a variation of ϵ_vac_ ranging from ∼2.08 eV to ∼2.13 eV (Figure 3b, yellow stars). The model predictions ϵ̂_vac_ again match quite well the target excitations ϵ_vac_ obtained through TD-DFT, despite the highly distorted geometries sampled along the scan coordinate (see, for example, Figure S2b).

**Figure 4:**
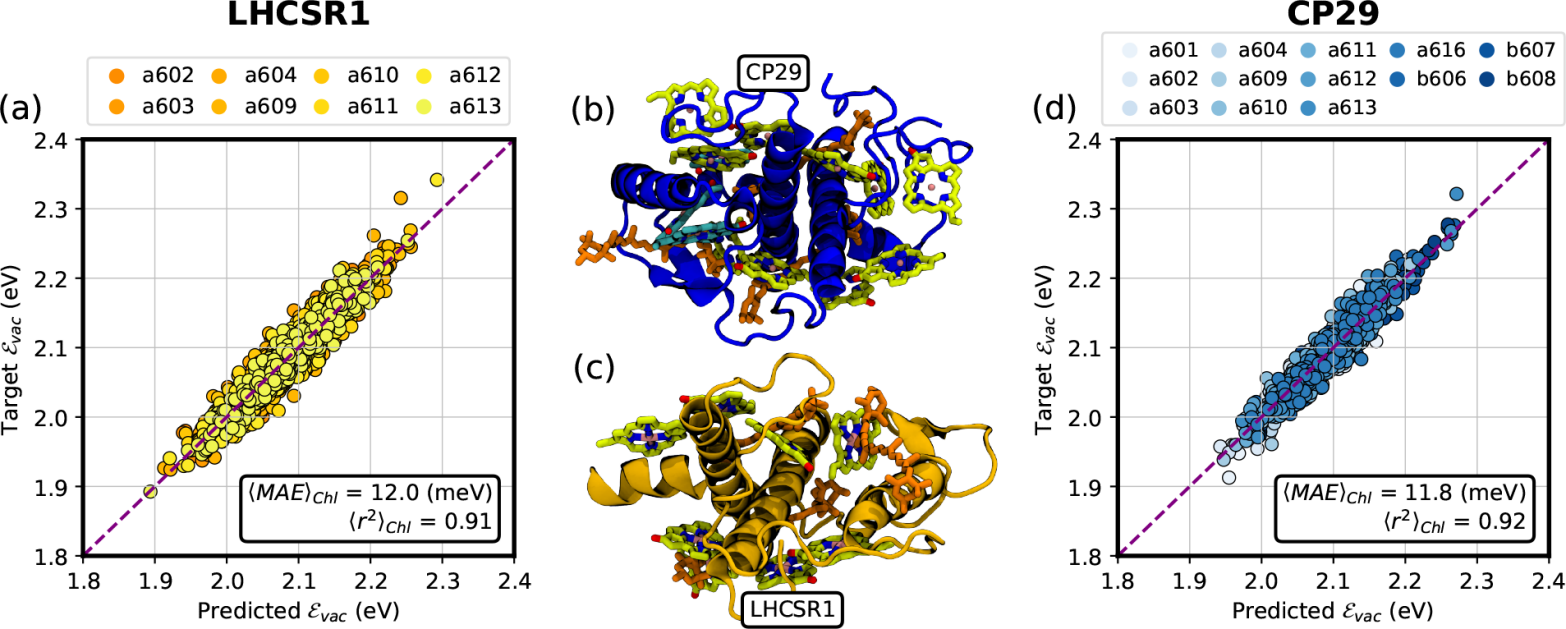
Performance of the vacuum ML model on different test sets. (a) Vacuum ML model predictions for chlorophylls *a* in LHCSR1. (b) Structure of CP29. Protein is shown in blue, Chls *a* are shown in green, Chls *b* are shown in cyan, and Cars are shown in orange. (c) Structure of LHCSR1. Protein is shown in yellow, Chls *a* are shown in green, and Cars are shown in orange. (d) Vacuum ML model predictions for chlorophylls *a* in CP29. In both panels (a) and (d), the inset reports the mean absolute error (MAE) and the Pearson’s *r*-squared, both averaged over the different chlorophylls.

As a final important test of the model, we have predicted the vacuum excitation energy ϵ̂_vac_ for two additional LHCs, namely LHCSR1 of algae and mosses and the minor LHC of higher plants CP29 (see Figure 4). We have employed the MD simulations of ref (44) to compute vacuum site energies of LHCSR1 at different frames, and the MD simulations of refs (50 and 52) for CP29. The LHCSR1 model contains 8 Chl *a*,^44^ while CP29 contains 10 Chl *a* and 3 Chl *b*,^53^ allowing us to test the model for both pigments.

The performance of the ML model on LHCSR1 and CP29 is shown in Figure 4a and d, respectively. In both cases, the *r*^2^ and MAE scores are in line with the predicted cross-validated scores (Figure 2). The average MAE on Chls *a* in LHCSR1 is ∼12 meV, while those of Chls *a* and *b* in CP29 are ∼12 and 11 meV, respectively. Note that the scores on Chl *a* and Chl *b* are lower than the best scores obtained in the learning curve on LHCII, because now the entire LHCII training set is employed to train the ML models. This test further confirms the reliability of the cross-validation estimates (Figure 2) and shows that vacuum site energies can be computed on Chl geometries other than those of LHCII, such as in different LHCs.

The good performance obtained on Chl geometries of practical interest, such as those of different LHCs, as well as on distorted Chl geometries, confirms that the ML model can reliably predict vacuum site energies accurately matching the TD-DFT ones.

### 4.2 Electrostatic Embedding ML Model

**Figure 5:**
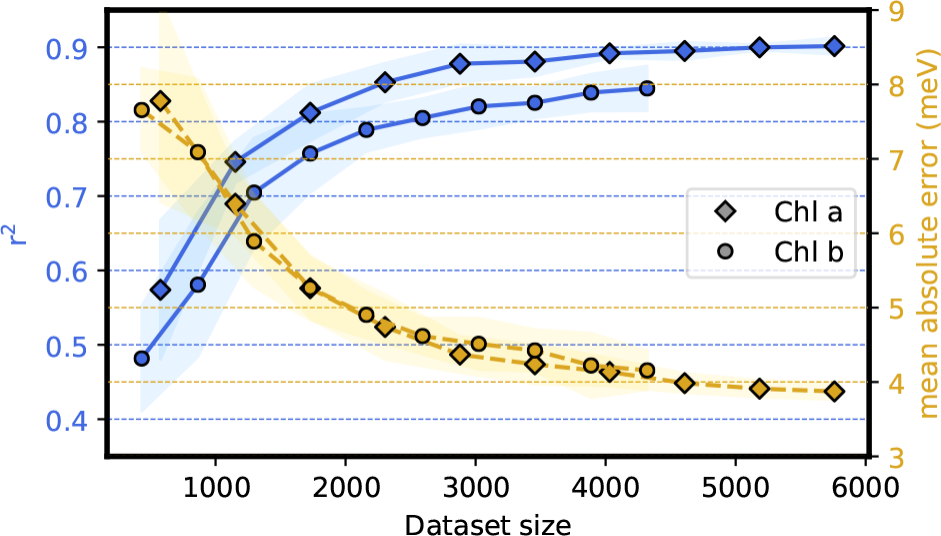
Learning curves for the electrochromic shift ϵ̂_shift_ of chlorophylls in LHCII. Blue lines report the Pearson’s *r*-squared, and yellow lines report the mean absolute error (MAE), both evaluated on the validation test with 5-fold cross-validation (CV-5). The uncertainty is computed as twice the standard deviation of the validation score and shown as a shaded region around the corresponding curve. The horizontal axis reports the dataset size used to perform CV-5. Diamond markers correspond to Chl *a*, while circles correspond to Chl *b*.

We now evaluate the performance and robustness of the EE ML model for the electrochromic shift. The learning curves for Chl *a* and Chl *b* are shown in Figure 5. At variance with the vacuum case (Figure 2), we observe the same learning pace for both Chl *a* and *b*, indicating that the model describes equally well the response of both pigments. We further note that convergence is reached more rapidly here than in the vacuum case, with a prediction error for both Chl *a* and Chl *b* approaching ∼4 meV. The improved rate of convergence of the EE ML model can be ascribed to the physical constraints that are built directly inside the kernel κ_shift_ (eq 12) and to the nature of the descriptor χ_Pot_ (eq 10) which transparently reflects the physics of the problem. We can appreciate the importance of incorporating the internal degrees of freedom into the model by testing a model that does not take the internal degrees of freedom into account. The learning curves in Figure S4 show that such a model would perform fairly worse, demonstrating the pivotal role of the pigment geometry in the response to the external potential.

As we have done for the vacuum ML model, we now consider more stringent tests of the model performance to assess the level of overfitting. In particular, we will determine if the model can be safely employed to predict site energies on other LHCs, and in general on arbitrary environments. We first test the EE ML model predictions on Chls of another LHC, the minor antenna CP29. We choose as our test set the following Chls: *a*609, which is a Chl *b* in LHCII;^53,54^*a*612, whose environment differs between LHCII and CP29;^49,52^*a*616, which is located near the flexible N-terminal and is characterized by a high static disorder in the MD simulation; and finally *b*606, to test the performance also on a Chl *b*. In addition, to compare with a well-established model, we have estimated the electrochromic shift using the charge density coupling (CDC) method.^55^ The CDC method employes fixed charges, representing the difference density Δρ upon excitation, to compute the electrochromic shift. It thus represents a “null model”, under the hypothesis that the electrochromic shift can be calculated from the properties of the isolated Chls.

**Figure 6:**
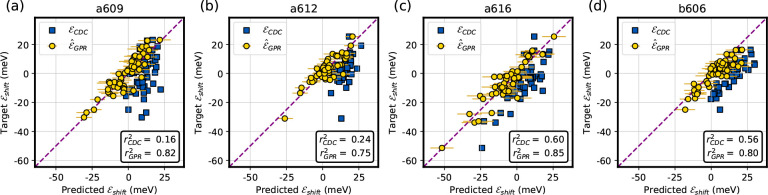
Prediction of the electrochromic shift ϵ_shift_ in Chls embedded in CP29. The prediction from the electrostatic embedding ML model (GPR) is shown in yellow circles, where for each point the model uncertainty, calculated as twice the square root of the posterior variance eq 6, is reported as a horizontal bar. Predictions from the charge density coupling (CDC) method are shown as blue squares. The Pearson’s *r*-squared is reported for each prediction. (a) Prediction on Chl *a*609 (MAE_GPR_ = 4.1 meV, MAE_CDC_ = 12.6 meV). (b) Prediction on Chl *a*612 (MAE_GPR_ = 3.7 meV, MAE_CDC_ = 12.7 meV). (c) Prediction on Chl *a*616 (MAE_GPR_ = 4.2 meV, MAE_CDC_ = 12.3 meV). (d) Prediction on Chl *b*606 (MAE_GPR_ = 4.2 meV, MAE_CDC_ = 14.0 meV).

The performance of the EE ML model in CP29 is shown in Figure 6. Despite the different environments experienced by the examined Chls, the ML model accurately predicts the TD-DFT electrochromic shifts. The error on this test set (MAE ≈ 4 meV) is similar to the error obtained by cross-validation, confirming that the model does not degrade when predicting outside the training dataset. The *r*^2^ scores are slightly lower than the cross-validated ones, due to the fact that here we considered each Chl separately, with a smaller dispersion of target values. Compared with the CDC method, our ML model shows a substantial improvement. In fact, the CDC method consistently shows smaller *r*^2^ values for the various Chls and an approximately 3-fold MAE. In addition, the CDC method seems systematically biased toward positive electrochromic shifts. While for *a*616 and *b*606 the CDC retains a correlation with the target data, for the other Chls its predictions are almost constant and uncorrelated with the target.

**Figure 7:**
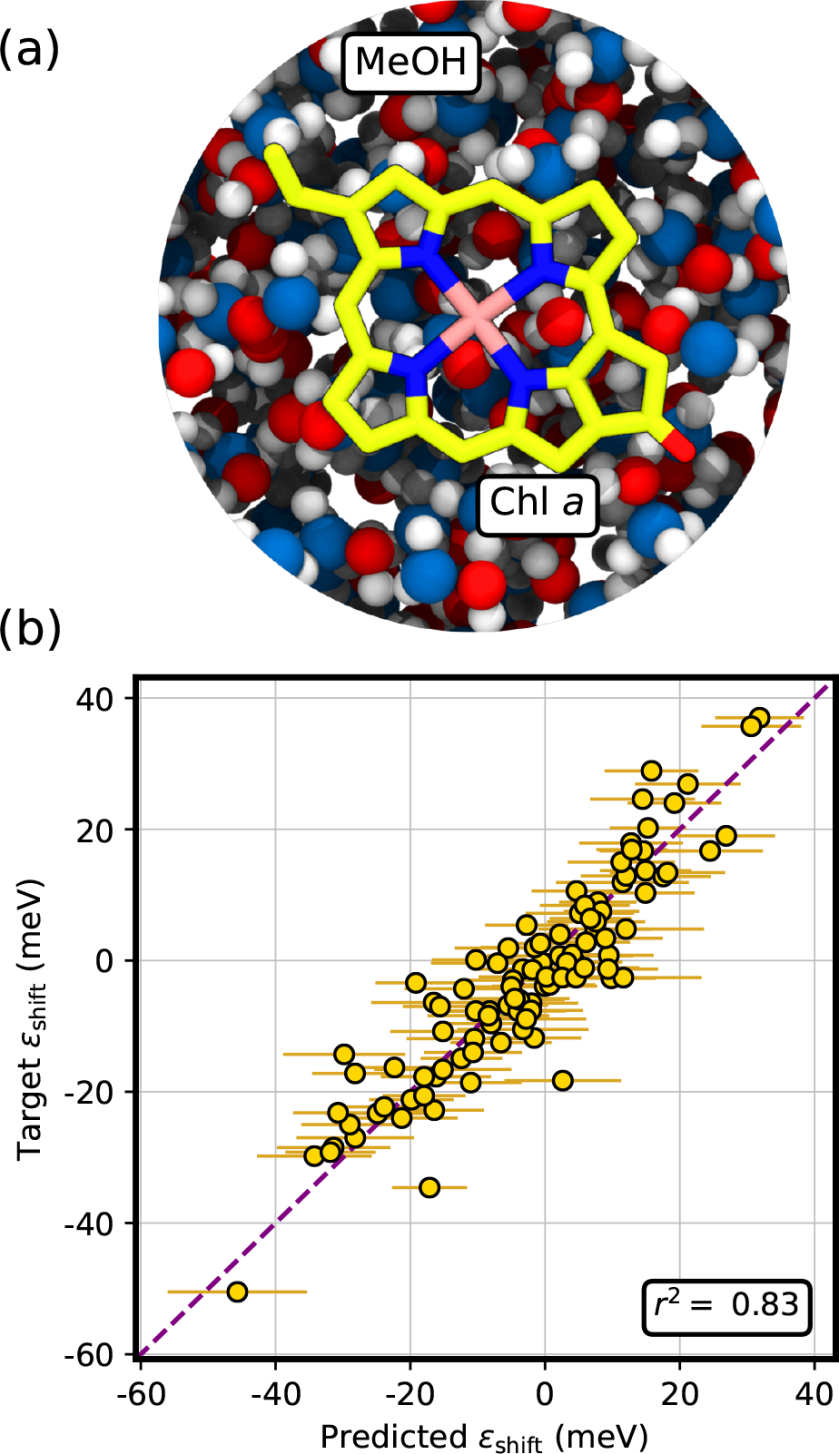
Estimation of the electrochromic shift ϵ_shift_ for Chl *a* embedded in methanol (see panel (a)). (b) Prediction of the electrostatic embedding ML model (GPR). The ML model uncertainty, computed as twice the square root of the posterior variance eq 6, is reported as a horizontal bar. The Pearson’s *r*-squared is reported in the inset. The corresponding mean absolute error (MAE) is 5.0 meV.

Our test set comprising Chls embedded in CP29 is an out-of-sample set, as the precise environment surrounding each Chl is different between the two LHCs. This confirms the reliability of the model on other pigment–protein complexes. However, the LHCII and CP29 environments are globally similar, both consisting of a protein matrix embedding the Chl, plus a lipid membrane and water molecules on both sides of the membrane. In order to test the model on even more out-of-sample configurations, we have predicted electrochromic shifts for Chl *a* in a polar solvent, methanol (Figure 7a). Geometries are sampled from a classical MD simulation, in order to thoroughly sample both the internal degrees of freedom of the Chl as well as the solvent ones. (More details on the classical MD simulation are provided in the Supporting Information.) As now the Chl is surrounded by a highly dynamic environment, rather than simply a protein pocket, we expect a larger variability in environment features which have not been seen by the model during the training.

Figure 7b shows the performance of our model for Chl *a* in methanol. We note that the *r*^2^ score (∼0.83) and the MAE (∼5 meV) are in good agreement both with the cross-validated ones and with those obtained for CP29. Importantly, the performance of the prediction does not degrade for positive ϵ_shift_ values, which do not appear in CP29 (Figure 6). This more stringent test shows that our model can correctly extrapolate well outside of the training set. This indicates that the EE ML model has not memorized the LHCII training set but instead has learned the correct physics underlying the electrochromic shift in a fully atomistic environment.

### 4.3 Polarizable ML Model

Having set up a model for the prediction of the electrochromic shift, we finally turn to the effect of polarization. The polarization contribution is not learned directly here, but it is approximated by eq 16. This allows us to exploit the prediction of transition charges developed in our previous work.^30^ We recall that by summing this term to the previous ones for ϵ_vac_ and the electrochromic shift ϵ_shift_, we finally obtain the site energy of the embedded chlorophyll.

**Figure 8:**
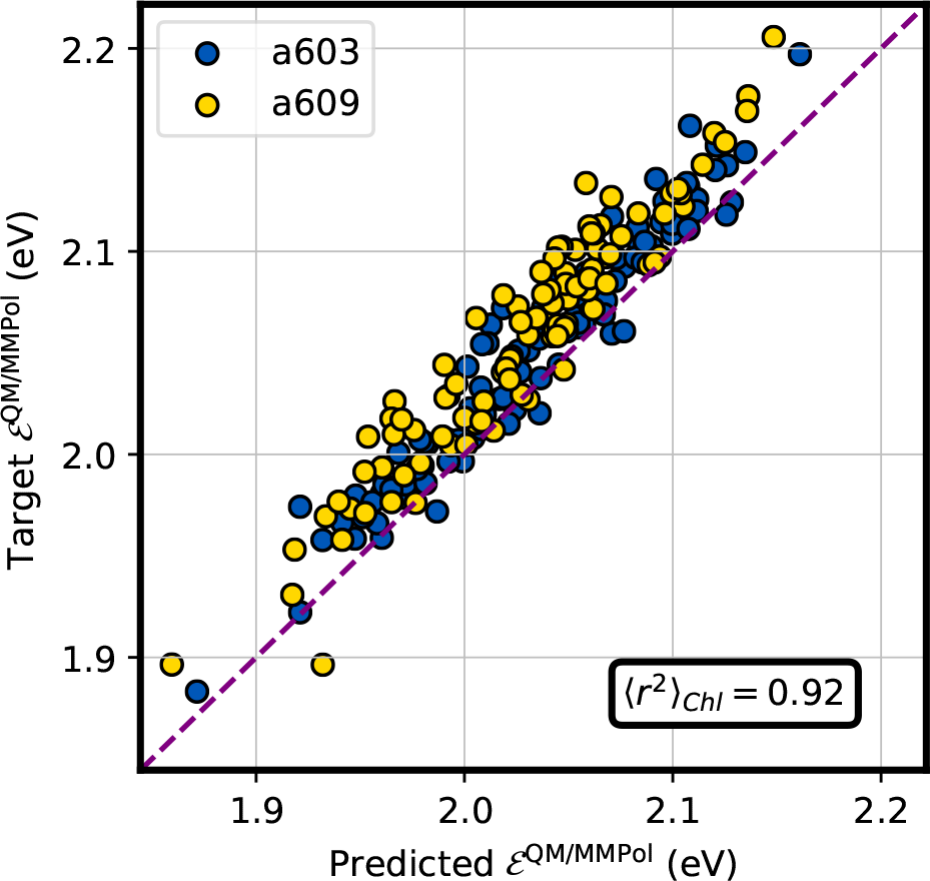
Performance of the polarizable embedding ML model. The prediction of the ML model is reported on the horizontal axis, and the target is reported on the vertical axis. Blue points correspond to Chl *a*603, and yellow points correspond to Chl *a*609. Both Chls belong to CP29. The Pearson’s *r*-squared averaged over the two Chls is reported in the inset.

In Figure 8 we compare the results of this prediction with the ones calculated at the TD-DFT QM/MMPol level for two different Chls (*a*603 and *a*609). The comparison shows a good agreement, and the *r*^2^ score of ∼0.92 shows that variations of the site energies are well captured by our polarizable ML model.

We note that the predicted site energy is shifted to lower values by a seemingly fixed amount compared to the target one (Figure 8), which translates into a MAE of ∼24.6 meV. This effect arises because in our ML sequential model we are neglecting the effect that the polarizable environment has on the transition charges that give rise to the polarization term. One possible way of accounting for this contribution would be to use effective transition charges *q*_eff_^tr^, which account for the alteration on the transition density when switching from an electrostatic embedding to a polarizable one. However, this deviation is essentially systematic, and in a first approximation it can be accounted for with a simple shift of the estimated site energy.

After having validated the ML models and demonstrated their accuracy and reliability in multiple contexts, we here showcase two applications of our ML estimation of Frenkel Hamiltonians.

### 4.4 Determining the Influence of Protein Residues

It is well known that one of the main roles of the protein in LHCs is to tune the energy levels of the embedded pigments through the electrostatic properties of their residues to optimize their function.^2,56^ Understanding how the protein residues influence the excitation properties of the chromophores is at the basis of a rational engineering of protein mutants with improved properties.^57,58^

In the first application, we show how to determine such an electrostatic influence of protein residues on the site energy of selected chlorophylls in LHCII. In this analysis we neglect the effect of a residue on the geometry of the pigment. This kind of estimation is useful to assess which residues are important for the spectral tuning of LHCs.^59^

**Figure 9:**
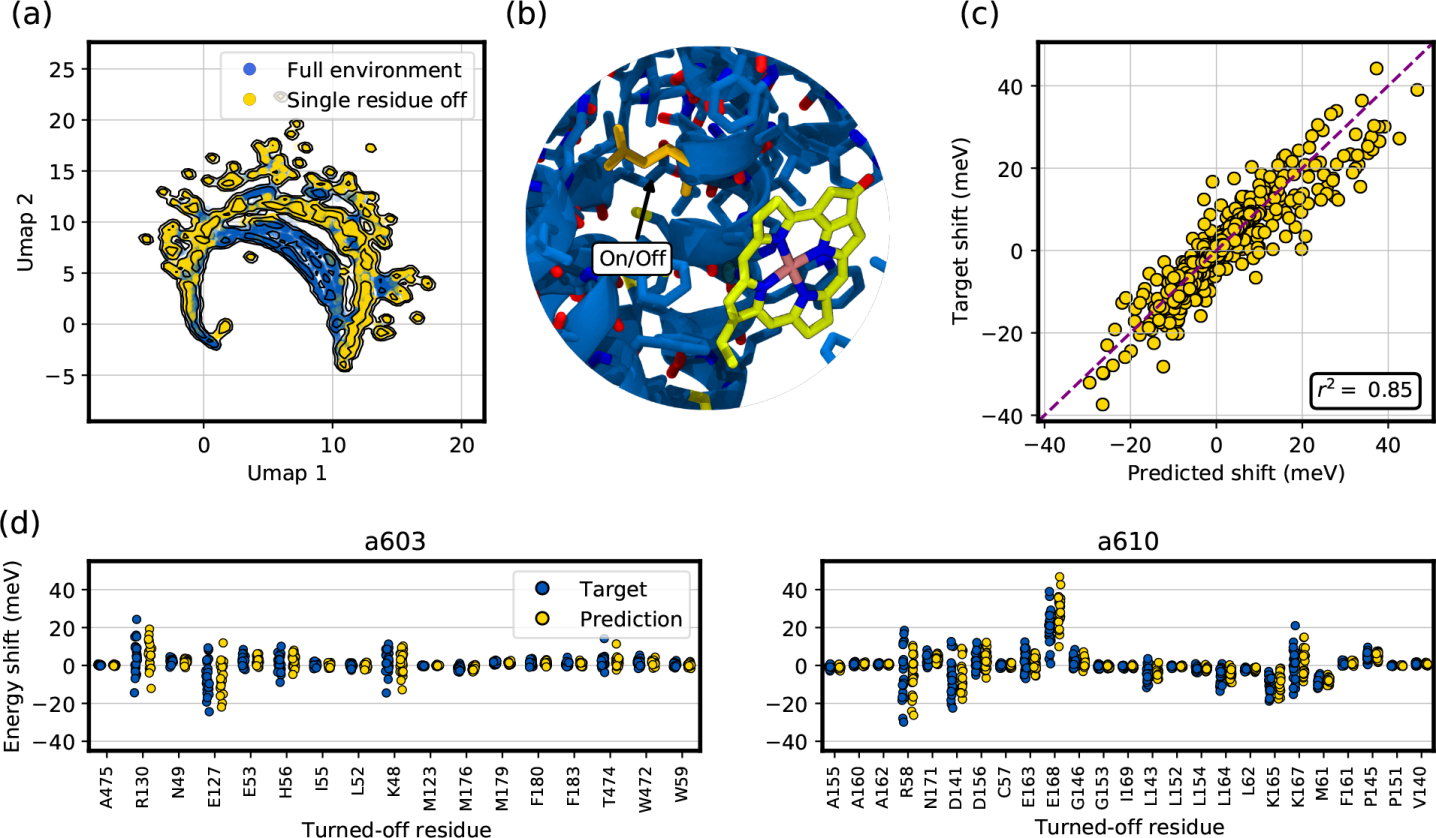
Excitation energy predictions when turning off the electrostatics of selected residues. (a) UMAP^60^ projection of the MM electrostatic potential on the QM atoms, when the environment comprises all the atoms (blue points) and when a single residue’s electrostatics is turned off (yellow points). (b) Illustration of the main idea. The effect of a given protein residue (depicted in yellow) on the excitation energy of a nearby Chl can be obtained by predicting the site energy with the residue’s electrostatics turned off. (c) Performance of the electrostatic embedding ML model in predicting the shift in site energy due to turning off the electrostatics-selected residues. The targets are the TD-DFT EE-QM/MM calculations. (d) Influence of each protein residue on tuning the site energy of Chl *a*603 (left) and Chl *a*610 (right). Blue points correspond to the target TD-DFT EE-QM/MM values, and yellow points are the ML model predictions.

The basic idea is illustrated in Figure 9b: the influence of residue *R* on the site energy of pigment *P* is computed by estimating the electrochromic shift twice: one when *R* contributes to the MM potential eq 10 felt by *P*, and one when *R* does not contribute to the potential (i.e., its electrostatics is turned off). The quantity ϵ_*P*:*R*_ = ϵ_*P*:*R*=on,shift_ – ϵ_*P*:*R*=off,shift_ quantifies the influence of residue *R* on the site energy of pigment *P*. Here ϵ_*P*:*R*=on,shift_ is the electrochromic shift computed when residue *R* is included in the MM potential acting on *P*, and ϵ_*P*:*R*=off,shift_ when *R* is not included.

Figure 9a shows the UMAP^60^ projection of the MM electrostatic potential when the electrostatics of nearby residues is left untouched (blue points) and when it is turned off (yellow points). It shows that, when turning off the electrostatics of a single residue, the MM potential felt by the QM system differs from what is usually present in the training set; i.e., we are slightly out of sample when predicting ϵ̂_*P*:*R*=off,shift_. For this reason, in addition of being an application of the ML models developed, the prediction of ϵ̂_*P*:*R*_ also serves as a further validation of the EE ML model.

The good performance of our EE ML model when estimating ϵ̂_*P*:*R*=off,shift_ is shown in Figure 9c, which shows the EE ML model prediction ϵ̂_*P*:*R*=off,shift_ against the target shift ϵ_*P*:*R*=off,shift_, as computed with TD-DFT M062X/6-31G(d) for some Chls, namely *a*603, *a*610, and *a*612 of different LHCII monomers. The high Pearson’s *r*-squared obtained (∼0.85) shows that the model can reliably estimate ϵ̂_*P*:*R*=off,shift_, enabling a rapid prediction of the influence of protein residues on the pigment excitation energies.

Figure 9d shows an example of the use of ϵ̂_*P*:*R*_ for two Chls. Here, residues located within 6 Å of the Chl are selected, and ϵ̂_*P*:*R*_ is computed with the EE ML model (yellow points). For each residue, we can estimate both its average effect and its dispersion. For example, E127 red-shifts the excitation of Chl *a*603 (Figure 9d, left), E168 blue-shifts the excitation of Chl *a*610 (Figure 9d, right), and L52 has virtually no influence on the excitation of Chl *a*603. Moreover, multiple residues (e.g., H56, K48 for Chl *a*603, and R58, D156, K167 for Chl *a*610) have a more complex effect on the Chl excitation, sometimes red-shifting and sometimes blue-shifting it, according to the protein conformation that is examined. These predictions are also compared with the target values ϵ_*P*:*R*_ (Figure 9d, blue points), showing that both the average and the spread of ϵ̂_*P*:*R*_ match those of the target, further proving the reliability of the model in estimating the shift.

We note that, contrary to the time required to compute ϵ_*P*:*R*_ with EE-QM/MM, the estimation of ϵ̂_*P*:*R*_ is extremely rapid. As such, it allows estimating, for example, the influence of each protein residue on the excitation energy of every Chl embedded in the protein, which would not be feasible in reasonable time with a straightforward QM/MM method.

### 4.5 Absorption Spectrum of CP29-WT and CP29-H111N

**Figure 10:**
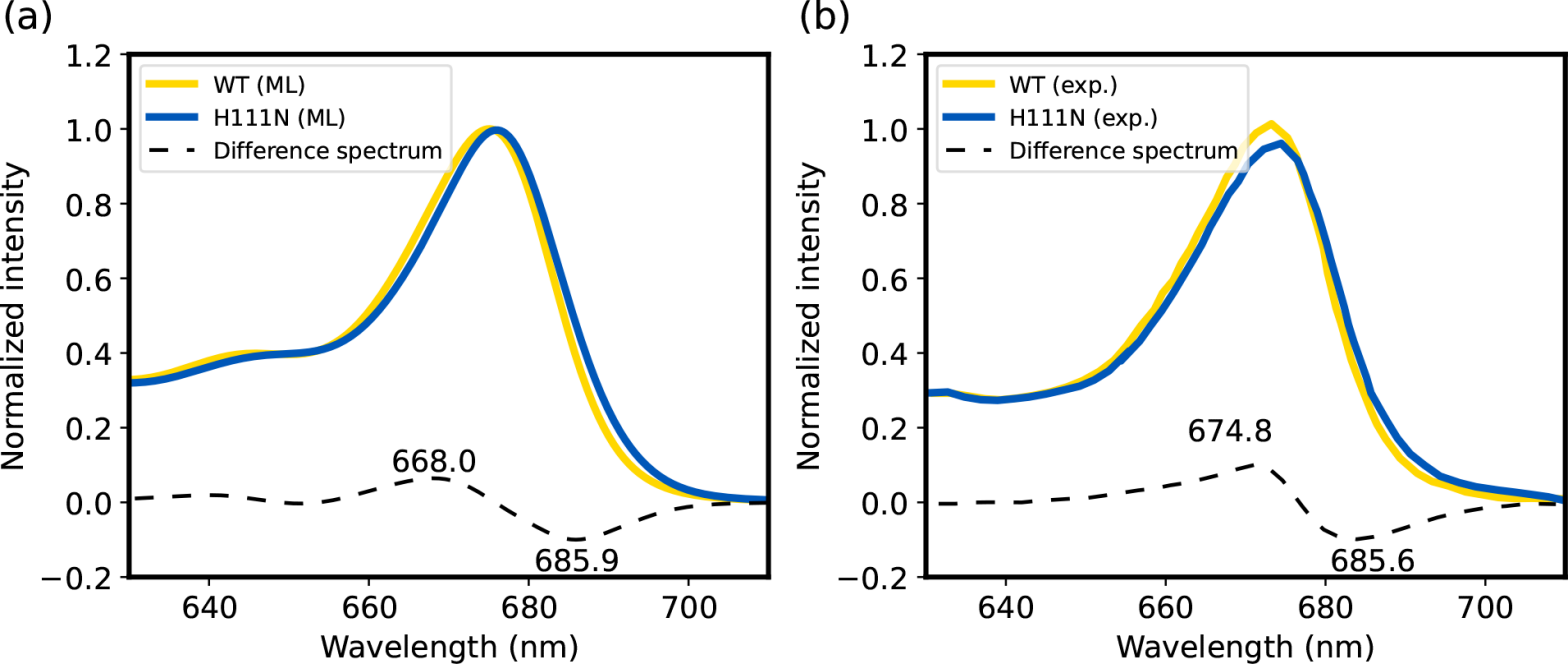
Absorption spectrum of CP29-WT and its mutant CP29-H111N. (a) Spectrum computed with our ML model, using the Full Cumulant Expansion formalism. (b) Experimental spectrum from ref (61). The spectrum of CP29-WT is shown in yellow, while the spectrum of CP29-H111N is shown in blue. The difference spectrum is reported as a black dashed line. The wavelengths of the minimum and maximum in the difference spectrum are reported.

As a second application, we showcase the ML-accelerated calculation of optical spectra for a whole LHC. We consider the minor LH complex CP29, and in particular the wild-type (WT) complex^53^ CP29-WT and its mutant CP29-H111N, where asparagine replaces H111, the axial ligand of Chl *a*603. Guardini et al.^61^ have shown that this pair of LHCs is particularly interesting, as the mutation induces an alteration of the local environment of Chl *a*603 which is reflected in the absorption spectrum^61^ (Figure 10b). We have previously confirmed their insights with MD simulations and QM/MMPol calculations.^7^

Our ML sequential strategy can be employed to obtain the very same quantitative estimates, with some key advantages. The computational cost is reduced by orders of magnitude, which means that we are not limited to characterize only the most important Chls, but instead the effect on all the other Chls can be estimated rapidly and with good accuracy. Furthermore, due to the reduced computational cost, we can obtain results that are far more statistically robust. A total of 6000 frames (3000 for CP29-WT, 3000 for CP29-H111N) have been employed, resulting in ∼78 000 site energies and ∼222 000 couplings. These calculations, including the polarization contribution, required approximately 3 days to complete on a single machine with four Intel Xeon Gold 5118 CPUs @2.30 GHz, while calculations excluding polarization required less than 3 h. As noted also in ref (30), the polarization contribution is the most expensive part: the EE ML model requires ∼0.1 s per calculation, while the polarizable ML model requires ∼3.5 s.

Figure S5 shows the site energies, as computed with the polarizable ML model herein developed, and the electronic couplings, computed with the model presented in ref (30), for CP29-WT and CP29-H111N. Our ML estimates confirm the increased coupling in the Chl *a*603–*a*609 pair^7,61^ and further show that smaller but significant effects are found for the coupling between Chls *a*603 and *a*616.

Finally, we have computed the absorption spectra of both CP29-WT (Figure 10a, yellow line) and CP29-H111N (Figure 10a, blue line), as well as their difference spectrum (Figure 10a, black dashed line). Details on the calculation of the absorption spectra are provided in the Supporting Information. The spectrum has been computed employing the Frenkel Hamiltonians estimated with our polarizable ML model, and computing the multichromophoric lineshape with the full cumulant expansion formalism.^62,63^ The computed spectrum reproduces well the experimental one^61^ (Figure 10b) and shows the two characteristic peaks in the difference mutant-minus-WT spectrum (∼668 nm and ∼686 nm in our estimate, ∼675 nm and ∼686 nm in the experimental spectrum). The estimated shift, which is slightly exaggerated compared to the experimental one, is compatible with the QM/MMPol one obtained in ref (7) with TD-DFT M062X/6-31G(d). This shows that our ML estimates can reliably be employed in models that start from excitonic Hamiltonians to produce spectra, with the same accuracy as the target QM method.

## 5 Conclusions

In this work, we have presented a ML-based strategy for the description of excitonic Hamiltonians of embedded multichromophoric systems along a molecular dynamics simulation. By building on the coupling model recently developed by us, here we complete the description by developing a Gaussian process ML model for the estimation of excitation energies including both electrostatic and polarization effects of the embedding environment.

We employed our model for the estimation of site energies of Chls *a* and *b* in light-harvesting complexes. While the model training was based on the LHCII complex of higher plants, the tests on different LHCs showed small errors and high correlation with the target excitation energies. The model trained on LHCII showed a remarkable performance also on an out-of-sample test case such as Chl *a* in methanol. The ability of our ML model to extrapolate to different cases indicates the robustness of our physics-based learning strategy. We also note that we trained our models on relatively small datasets, demonstrating that a quite good accuracy can be obtained with a reasonable number of QM calculations.

The utility of our ML model has been showcased in two examples: First, we obtained a fast estimation of the effect of protein residues on the site energy of the Chls, which opens up the possibility to quickly determine the importance of each residue in the spectral tuning of the chromophore’s excitation. Then we computed exciton Hamiltonians for 3000 MD frames of the wild-type CP29 complex and its H111N mutant. This allowed us to accurately compute the absorption spectra for the two complexes and compare the difference spectrum with the experiments, reproducing the experimental results.

The ML models presented here can help with computing excitation energies and couplings in LHCs different from the ones analyzed here, with considerable accuracy and time savings. Indeed, LHCs are bound to many chromophores, and relying on QM calculations to compute exciton properties is too expensive to obtain proper statistics. Furthermore, as conformational changes of LHCs are connected to their function, having a fast method to compute exciton properties of these complexes comes in handy when analyzing multiple conformations from MD simulations, e.g., by connecting specific conformations to weakened or enhanced interactions of the chromophores. As we have showcased in the case of a particular LHC mutant, our models also provide a quantitative way to rapidly screen the excitation properties of mutants. This makes it possible to analyze known mutants as well as providing a rational basis with which mutants can be devised *in silico*, i.e., by inserting mutations and then rapidly assessing their impact on the exciton properties of the bound pigments. Another interesting application to be explored in the future is the ML determination of spectral densities from QM/MM trajectories.^15,64^

This approach provides a fast and accurate estimation of excitonic Hamiltonians for an arbitrary number of MD structures. While in this work we focused on Chls *a* and *b* in light-harvesting complexes, the remarkable performance on several tests makes our model a promising tool for accelerating calculations also for other protein-embedded chromophores. Finally, the learning approach showcased here is by no means limited to light-harvesting complexes but can be employed in far more general settings.

## Supporting Information Available

Analysis of the performance of different ML models and descriptors in a vacuum; analysis of the conformational freedom of Chls *a* and *b*; details on the scan over an improper dihedral of Chl *a*; analysis of the importance of internal coordinates in the EE ML model; details on the MD of Chl *a* in methanol; details on site energies and couplings predicted with the ML models in CP29-WT and CP29-H111N; details on the calculation of the absorption spectrum in CP29-WT and CP29-H111N (PDF)

## References

[ref1] Scholes G. D.; Fleming G. R.; Olaya-Castro A.; van Grondelle R. Lessons from nature about solar light harvesting. Nat. Chem. 2011, 3, 763–774. 10.1038/nchem.1145. 21941248

[ref2] Croce R.; van Amerongen H. Natural strategies for photosynthetic light harvesting. Nat. Chem. Biol. 2014, 10, 492–501. 10.1038/nchembio.1555. 24937067

[ref3] Mirkovic T.; Ostroumov E. E.; Anna J. M.; van Grondelle R.; Govindjee; Scholes G. D. Light Absorption and Energy Transfer in the Antenna Complexes of Photosynthetic Organisms. Chem. Rev. 2017, 117, 249–293. 10.1021/acs.chemrev.6b00002. 27428615

[ref4] Abramavicius D.; Butkus V.; Valkunas L.. Semiconductors and Semimetals; Elsevier, 2011; Vol. 85, pp 3–46.

[ref5] Lambrev P. H.; Akhtar P.; Tan H.-S. Insights into the mechanisms and dynamics of energy transfer in plant light-harvesting complexes from two-dimensional electronic spectroscopy. Biochim. Biophys. Acta, Bioenerg. 2020, 1861, 148050 10.1016/j.bbabio.2019.07.005. 31326408

[ref6] Jansen T. L. C. Computational spectroscopy of complex systems. J. Chem. Phys. 2021, 155, 170901 10.1063/5.0064092. 34742221

[ref7] Cignoni E.; Slama V.; Cupellini L.; Mennucci B. The atomistic modeling of light-harvesting complexes from the physical models to the computational protocol. J. Chem. Phys. 2022, 156, 120901 10.1063/5.0086275. 35364859

[ref8] Chenu A.; Scholes G. D. Coherence in Energy Transfer and Photosynthesis. Annu. Rev. Phys. Chem. 2015, 66, 69–96. 10.1146/annurev-physchem-040214-121713. 25493715

[ref9] Jang S. J.; Mennucci B. Delocalized excitons in natural light-harvesting complexes. Rev. Mod. Phys. 2018, 90, 035003 10.1103/RevModPhys.90.035003.

[ref10] Curutchet C.; Mennucci B. Quantum Chemical Studies of Light Harvesting. Chem. Rev. 2017, 117, 294–343. 10.1021/acs.chemrev.5b00700. 26958698

[ref11] Cupellini L.; Bondanza M.; Nottoli M.; Mennucci B. Successes & challenges in the atomistic modeling of light-harvesting and its photoregulation. Biochim. Biophys. Acta - Bioenerg. 2020, 1861, 148049 10.1016/j.bbabio.2019.07.004. 31386831

[ref12] Segatta F.; Cupellini L.; Garavelli M.; Mennucci B. Quantum Chemical Modeling of the Photoinduced Activity of Multichromophoric Biosystems. Chem. Rev. 2019, 119, 9361–9380. 10.1021/acs.chemrev.9b00135. 31276384 PMC6716121

[ref13] Maity S.; Kleinekathöfer U. Recent progress in atomistic modeling of light-harvesting complexes: a mini review. Photosynth. Res. 2022, 10.1007/s11120-022-00969-w. PMC10070314 36207489

[ref14] Maity S.; Daskalakis V.; Elstner M.; Kleinekathöfer U. Multiscale QM/MM molecular dynamics simulations of the trimeric major light-harvesting complex II. Phys. Chem. Chem. Phys. 2021, 23, 7407–7417. 10.1039/D1CP01011E. 33876100

[ref15] Sarngadharan P.; Maity S.; Kleinekathöfer U. Spectral densities and absorption spectra of the core antenna complex CP43 from photosystem II. J. Chem. Phys. 2022, 156, 215101 10.1063/5.0091005. 35676138

[ref16] Bondanza M.; Nottoli M.; Cupellini L.; Lipparini F.; Mennucci B. Polarizable embedding QM/MM: the future gold standard for complex (bio)systems?. Phys. Chem. Chem. Phys. 2020, 22, 14433–14448. 10.1039/D0CP02119A. 32588851

[ref17] Wang C.-I.; Joanito I.; Lan C.-F.; Hsu C.-P. Artificial neural networks for predicting charge transfer coupling. J. Chem. Phys. 2020, 153, 214113 10.1063/5.0023697. 33291923

[ref18] Krämer M.; Dohmen P. M.; Xie W.; Holub D.; Christensen A. S.; Elstner M. Charge and Exciton Transfer Simulations Using Machine-Learned Hamiltonians. J. Chem. Theory Comput. 2020, 16, 4061–4070. 10.1021/acs.jctc.0c00246. 32491856

[ref19] Farahvash A.; Lee C.-K.; Sun Q.; Shi L.; Willard A. P. Machine learning Frenkel Hamiltonian parameters to accelerate simulations of exciton dynamics. J. Chem. Phys. 2020, 153, 074111 10.1063/5.0016009. 32828098

[ref20] Chen Z.; Bononi F. C.; Sievers C. A.; Kong W.-Y.; Donadio D. UV–Visible Absorption Spectra of Solvated Molecules by Quantum Chemical Machine Learning. J. Chem. Theory Comput. 2022, 18, 4891–4902. 10.1021/acs.jctc.1c01181. 35913220

[ref21] Häse F.; Valleau S.; Pyzer-Knapp E.; Aspuru-Guzik A. Machine learning exciton dynamics. Chem. Sci. 2016, 7, 5139–5147. 10.1039/C5SC04786B. 30155164 PMC6020119

[ref22] Chen M. S.; Zuehlsdorff T. J.; Morawietz T.; Isborn C. M.; Markland T. E. Exploiting Machine Learning to Efficiently Predict Multidimensional Optical Spectra in Complex Environments. J. Phys. Chem. Lett. 2020, 11, 7559–7568. 10.1021/acs.jpclett.0c02168. 32808797

[ref23] Zeng J.; Giese T. J.; Ekesan S.; York D. M. Development of Range-Corrected Deep Learning Potentials for Fast, Accurate Quantum Mechanical/Molecular Mechanical Simulations of Chemical Reactions in Solution. J. Chem. Theory Comput. 2021, 17, 6993–7009. 10.1021/acs.jctc.1c00201. 34644071 PMC8578402

[ref24] Pan X.; Yang J.; Van R.; Epifanovsky E.; Ho J.; Huang J.; Pu J.; Mei Y.; Nam K.; Shao Y. Machine-Learning-Assisted Free Energy Simulation of Solution-Phase and Enzyme Reactions. J. Chem. Theory Comput. 2021, 17, 5745–5758. 10.1021/acs.jctc.1c00565. 34468138 PMC9070000

[ref25] Gastegger M.; Schütt K. T.; Müller K.-R. Machine learning of solvent effects on molecular spectra and reactions. Chem. Sci. 2021, 12, 11473–11483. 10.1039/D1SC02742E. 34567501 PMC8409491

[ref26] Shen L.; Wu J.; Yang W. Multiscale Quantum Mechanics/Molecular Mechanics Simulations with Neural Networks. J. Chem. Theory Comput. 2016, 12, 4934–4946. 10.1021/acs.jctc.6b00663. 27552235 PMC6209101

[ref27] Shen L.; Yang W. Molecular Dynamics Simulations with Quantum Mechanics/Molecular Mechanics and Adaptive Neural Networks. J. Chem. Theory Comput. 2018, 14, 1442–1455. 10.1021/acs.jctc.7b01195. 29438614 PMC6233882

[ref28] Böselt L.; Thürlemann M.; Riniker S. Machine Learning in QM/MM Molecular Dynamics Simulations of Condensed-Phase Systems. J. Chem. Theory Comput. 2021, 17, 2641–2658. 10.1021/acs.jctc.0c01112. 33818085

[ref29] Zinovjev K. Electrostatic Embedding of Machine Learning Potentials. ChemRxiv.org ePrint archive 2022, 10.26434/chemrxiv-2022-rknwt-v3. PMC10061678 36821513

[ref30] Cignoni E.; Cupellini L.; Mennucci B. A fast method for electronic couplings in embedded multichromophoric systems. J. Phys.: Condens. Matter 2022, 34, 304004 10.1088/1361-648X/ac6f3c. 35552268

[ref31] Rasmussen C. E.; Williams C. K. I. Gaussian processes for machine learning; MIT Press, 2005.

[ref32] Deringer V. L.; Bartók A. P.; Bernstein N.; Wilkins D. M.; Ceriotti M.; Csányi G. Gaussian Process Regression for Materials and Molecules. Chem. Rev. 2021, 121, 10073–10141. 10.1021/acs.chemrev.1c00022. 34398616 PMC8391963

[ref33] Cignoni E.; Cupellini L.; Mennucci B. excipy: Machine learning models for a fast estimation of excitonic Hamiltonians. Zenodo 2023, 10.5281/zenodo.7503183.

[ref34] Cupellini L.; Corbella M.; Mennucci B.; Curutchet C. Electronic energy transfer in biomacromolecules. WIREs Comput. Mol. Sci. 2019, 9, e1392 10.1002/wcms.1392.

[ref35] Christianen A.; Karman T.; Vargas-Hernández R. A.; Groenenboom G. C.; Krems R. V. Six-dimensional potential energy surface for NaK–NaK collisions: Gaussian process representation with correct asymptotic form. J. Chem. Phys. 2019, 150, 064106 10.1063/1.5082740. 30770001

[ref36] Dral P. O.; Barbatti M. Molecular excited states through a machine learning lens. Nat. Rev. Chem. 2021, 5, 388–405. 10.1038/s41570-021-00278-1. 37118026

[ref37] Westermayr J.; Marquetand P. Machine learning and excited-state molecular dynamics. Mach. Learn.: Sci. Technol. 2020, 1, 043001 10.1088/2632-2153/ab9c3e.

[ref38] Rupp M.; Tkatchenko A.; Müller K.-R.; von Lilienfeld O. A. Fast and Accurate Modeling of Molecular Atomization Energies with Machine Learning. Phys. Rev. Lett. 2012, 108, 058301 10.1103/PhysRevLett.108.058301. 22400967

[ref39] Montavon G.; Rupp M.; Gobre V.; Vazquez-Mayagoitia A.; Hansen K.; Tkatchenko A.; Müller K.-R.; Anatole von Lilienfeld O. Machine learning of molecular electronic properties in chemical compound space. New J. Phys. 2013, 15, 095003 10.1088/1367-2630/15/9/095003.

[ref40] Nottoli M.; Cupellini L.; Lipparini F.; Granucci G.; Mennucci B. Multiscale Models for Light-Driven Processes. Annu. Rev. Phys. Chem. 2021, 72, 489–513. 10.1146/annurev-physchem-090419-104031. 33561359

[ref41] Corni S.; Cammi R.; Mennucci B.; Tomasi J. Electronic excitation energies of molecules in solution within continuum solvation models: investigating the discrepancy between state-specific and linear-response methods. J. Chem. Phys. 2005, 123, 134512 10.1063/1.2039077. 16223319

[ref42] Madjet M. E.; Abdurahman A.; Renger T. Intermolecular Coulomb Couplings from Ab Initio Electrostatic Potentials: Application to Optical Transitions of Strongly Coupled Pigments in Photosynthetic Antennae and Reaction Centers. J. Phys. Chem. B 2006, 110, 17268–17281. 10.1021/jp0615398. 16928026

[ref43] Slama V.; Cupellini L.; Mennucci B. Exciton properties and optical spectra of light harvesting complex II from a fully atomistic description. Phys. Chem. Chem. Phys. 2020, 22, 16783–16795. 10.1039/D0CP02492A. 32662461

[ref44] Guarnetti Prandi I.; Sláma V.; Pecorilla C.; Cupellini L.; Mennucci B. Structure of the stress-related LHCSR1 complex determined by an integrated computational strategy. Commun. Biol. 2022, 5, 145 10.1038/s42003-022-03083-8. 35177775 PMC8854571

[ref45] Hornak V.; Abel R.; Okur A.; Strockbine B.; Roitberg A.; Simmerling C. Comparison of multiple Amber force fields and development of improved protein backbone parameters. Proteins Struct. Funct. Bioinform. 2006, 65, 712–725. 10.1002/prot.21123. PMC4805110 16981200

[ref46] Wang J.; Cieplak P.; Li J.; Hou T.; Luo R.; Duan Y. Development of Polarizable Models for Molecular Mechanical Calculations I: Parameterization of Atomic Polarizability. J. Phys. Chem. B 2011, 115, 3091–3099. 10.1021/jp112133g. 21391553 PMC3082581

[ref47] Frisch M. J.; Trucks G. W.; Schlegel H. B.; Scuseria G. E.; Robb M. A.; Cheeseman J. R.; Scalmani G.; Barone V.; Petersson G. A.; Nakatsuji H.; Li X.; Caricato M.; Marenich A. V.; Bloino J.; Janesko B. G.; Gomperts R.; Mennucci B.; Hratchian H. P.; Ortiz J. V.; Izmaylov A. F.; Sonnenberg J. L.; Williams- Young D.; Ding F.; Lipparini F.; Egidi F.; Goings J.; Peng B.; Petrone A.; Henderson T.; Ranasinghe D.; Zakrzewski V. G.; Gao J.; Rega N.; Zheng G.; Liang W.; Hada M.; Ehara M.; Toyota K.; Fukuda R.; Hasegawa J.; Ishida M.; Nakajima T.; Honda Y.; Kitao O.; Nakai H.; Vreven T.; Throssell K.; Montgomery J. A. Jr.; Peralta J. E.; Ogliaro F.; Bearpark M. J.; Heyd J. J.; Brothers E. N.; Kudin K. N.; Staroverov V. N.; Keith T. A.; Kobayashi R.; Normand J.; Raghavachari K.; Rendell A. P.; Burant J. C.; Iyengar S. S.; Tomasi J.; Cossi M.; Millam J. M.; Klene M.; Adamo C.; Cammi R.; Ochterski J. W.; Martin R. L.; Morokuma K.; Farkas O.; Foresman J. B.; Fox D. J. Gaussian 16, Revision A.03; Gaussian Inc.: Wallingford, CT, 2016.

[ref48] Balevičius V.; Fox K. F.; Bricker W. P.; Jurinovich S.; Prandi I. G.; Mennucci B.; Duffy C. D. P. Fine control of chlorophyll-carotenoid interactions defines the functionality of light-harvesting proteins in plants. Sci. Rep. 2017, 7, 13956 10.1038/s41598-017-13720-6. 29066753 PMC5655323

[ref49] Cupellini L.; Calvani D.; Jacquemin D.; Mennucci B. Charge transfer from the carotenoid can quench chlorophyll excitation in antenna complexes of plants. Nat. Commun. 2020, 11, 662 10.1038/s41467-020-14488-6. 32005811 PMC6994720

[ref50] Lapillo M.; Cignoni E.; Cupellini L.; Mennucci B. The energy transfer model of nonphotochemical quenching: Lessons from the minor CP29 antenna complex of plants. Biochim. Biophys. Acta, Bioenerg. 2020, 1861, 148282 10.1016/j.bbabio.2020.148282. 32721398

[ref51] Imbalzano G.; Anelli A.; Giofré D.; Klees S.; Behler J.; Ceriotti M. Automatic selection of atomic fingerprints and reference configurations for machine-learning potentials. J. Chem. Phys. 2018, 148, 241730 10.1063/1.5024611. 29960368

[ref52] Cignoni E.; Lapillo M.; Cupellini L.; Acosta-Gutiérrez S.; Gervasio F. L.; Mennucci B. A different perspective for nonphotochemical quenching in plant antenna complexes. Nat. Commun. 2021, 12, 7152 10.1038/s41467-021-27526-8. 34887401 PMC8660843

[ref53] Wei X.; Su X.; Cao P.; Liu X.; Chang W.; Li M.; Zhang X.; Liu Z. Structure of spinach photosystem II–LHCII supercomplex at 3.2 Åresolution. Nature 2016, 534, 69–74. 10.1038/nature18020. 27251276

[ref54] Liu Z.; Yan H.; Wang K.; Kuang T.; Zhang J.; Gui L.; An X.; Chang W. Crystal structure of spinach major light-harvesting complex at 2.72 resolution. Nature 2004, 428, 287–292. 10.1038/nature02373. 15029188

[ref55] Adolphs J.; Müh F.; Madjet M. E.-A.; Renger T. Calculation of pigment transition energies in the FMO protein: From simplicity to complexity and back. Photosynth Res. 2008, 95, 197–209. 10.1007/s11120-007-9248-z. 17917787

[ref56] Curutchet C.; Kongsted J.; Muñoz-Losa A.; Hossein-Nejad H.; Scholes G. D.; Mennucci B. Photosynthetic Light-Harvesting Is Tuned by the Heterogeneous Polarizable Environment of the Protein. J. Am. Chem. Soc. 2011, 133, 3078–3084. 10.1021/ja110053y. 21322565

[ref57] Blankenship R. E.; Tiede D. M.; Barber J.; Brudvig G. W.; Fleming G.; Ghirardi M.; Gunner M. R.; Junge W.; Kramer D. M.; Melis A.; Moore T. A.; Moser C. C.; Nocera D. G.; Nozik A. J.; Ort D. R.; Parson W. W.; Prince R. C.; Sayre R. T. Comparing Photosynthetic and Photovoltaic Efficiencies and Recognizing the Potential for Improvement. Science 2011, 332, 805–809. 10.1126/science.1200165. 21566184

[ref58] Srivastava A.; Ahad S.; Wat J. H.; Reppert M. Accurate prediction of mutation-induced frequency shifts in chlorophyll proteins with a simple electrostatic model. J. Chem. Phys. 2021, 155, 151102 10.1063/5.0064567. 34686046

[ref59] Ramos F. C.; Nottoli M.; Cupellini L.; Mennucci B. The molecular mechanisms of light adaption in light-harvesting complexes of purple bacteria revealed by a multiscale modeling. Chem. Sci. 2019, 10, 9650–9662. 10.1039/C9SC02886B. 32055335 PMC6988754

[ref60] McInnes L.; Healy J.; Melville J. UMAP: Uniform Manifold Approximation and Projection for Dimension Reduction. arXiv.org ePrint Archive 2020, arXiv:1802.03426 10.48550/arXiv.1802.03426.

[ref61] Guardini Z.; Bressan M.; Caferri R.; Bassi R.; Dall’Osto L. Identification of a pigment cluster catalysing fast photoprotective quenching response in CP29. Nat. Plants 2020, 6, 303–313. 10.1038/s41477-020-0612-8. 32170280

[ref62] Cupellini L.; Lipparini F.; Cao J. Absorption and Circular Dichroism Spectra of Molecular Aggregates With the Full Cumulant Expansion. J. Phys. Chem. B 2020, 124, 8610–8617. 10.1021/acs.jpcb.0c05180. 32901476 PMC7901647

[ref63] Ma J.; Cao J. Förster resonance energy transfer, absorption and emission spectra in multichromophoric systems. I. Full cumulant expansions and system-bath entanglement. J. Chem. Phys. 2015, 142, 094106 10.1063/1.4908599. 25747060

[ref64] Maity S.; Sarngadharan P.; Daskalakis V.; Kleinekathöfer U. Time-dependent atomistic simulations of the CP29 light-harvesting complex. J. Chem. Phys. 2021, 155, 055103 10.1063/5.0053259. 34364345

