# Supplementary material for: Machine Learning Exciton Hamiltonians in Light-Harvesting Complexes
Source: J Chem Theory Comput. 2023 Jan 26;19(3):965–77. doi: 10.1021/acs.jctc.2c01044 (PMC9933434; doi:10.1021/acs.jctc.2c01044)
Supplement: Supplementary file 1 — ct2c01044_si_001.pdf [file ct2c01044_si_001.pdf]

Supporting Information: Machine Learning Excitonic Parameters in Light Harvesting Complexes

Edoardo Cignoni,* Lorenzo Cupellini,* and Benedetta Mennucci

Dipartimento di Chimica e Chimica Industriale, University of Pisa, via G. Moruzzi 13, 56124, Pisa, Italy

E-mail: edoardo.cignoni@phd.unipi.it; lorenzo.cupellini@unipi.it

Machine Learning Models of Excitation Energies in Vacuum

We have tested different machine learning models and descriptors for predicting the vacuum excitation energy. For simplicity, we have limited our tests to the prediction of the vacuum excitation energy of Chl *a*.

We have employed two descriptors of the internal geometry of the chlorophyll: the Coulomb Matrix[S1] and a descriptor that encodes the molecular geometry as a list of bonds, angles, and dihedrals, similar to what is done in Ref. S2. In order to account for the dihedral periodicity, we have encoded each dihedral ϕ as a $\{\cos(\phi), \sin(\phi)\}$ pair. We refer to this descriptor as Bond-Angle-Dihedral Matrix (BADM).

We have also tested different kernels for the Gaussian Process regression, and a simple multilayer perceptron (MLP) neural network (NN). All Gaussian Process models are trained with the limited-memory Broyden–Fletcher–Goldfarb–Shanno (L-BFGS) algorithm maxi-

Table S1: Performance of different machine learning models on the prediction of the excitation energy (Q_y) of Chl *a*. BADM: Bond-Angle-Dihedral Matrix. CM: Coulomb Matrix. MLP: multilayer perceptron. Matern(x): Gaussian Process with Matern kernel ($\nu = x$). Squared Exponential: Gaussian Process with squared exponential kernel. Linear: Gaussian Process with linear kernel. MAE: mean absolute error. R^2: squared Pearson's R. All the results are obtained with five-fold cross-validation (CV-5). Values are provided as $\mu \pm 2\sigma$, where μ is the mean of the five CV folds, and σ is the corresponding standard deviation.

Descriptor	Model/Kernel	MAE (meV)	R^2	Train time (s)
BADM	MLP	17.6 ± 0.7	0.82 ± 0.04	81 ± 14
BADM	Matern(5/2)	13.1 ± 0.4	0.89 ± 0.00	10 ± 3
BADM	Matern(3/2)	13.2 ± 0.6	0.89 ± 0.01	12 ± 4
BADM	Squared Exponential	12.9 ± 0.4	0.90 ± 0.02	11 ± 3
BADM	Linear	12.8 ± 1.1	0.90 ± 0.02	5 ± 2
CM	Matern(5/2)	12.7 ± 0.9	0.91 ± 0.01	12 ± 4
CM	Matern(3/2)	13.6 ± 0.6	0.89 ± 0.02	15 ± 3
CM	Squared Exponential	12.3 ± 0.3	0.91 ± 0.01	10 ± 3
CM	Linear	13.6 ± 0.9	0.89 ± 0.01	4 ± 2
CM	MLP	17.0 ± 1.8	0.83 ± 0.03	200 ± 230

mizing the marginal log-likelihood. All Gaussian Process models have been implemented in GPflow.[S3] We have built the MLP with the same architecture presented in Ref. S4, namely with two hidden layers of 204 and 192 neurons, with the difference that we have used a Rectified Linear Unit as activation function. We have trained the MLP with the Adam optimizer, using a learning rate of 10^{-4} and L_2 regularization of 10^{-4}. A learning rate scheduler was employed to lower the learning rate each time the validation mean squared error reached a plateau, i.e., if for 400 consecutive steps the validation error was above the previous value plus an offset of 10^{-6}. In order to avoid overfitting, we have used early stopping, monitoring the validation mean squared error with a patience parameter of 1000 steps. Finally, in order to stabilize gradients in the first steps, we have clipped the norm of the gradients to 10^{-3}. The maximum number of epochs employed was $2 \cdot 10^5$, after which the training was stopped. The MLP model and training have been implemented in PyTorch.[S5] All models are trained using a Tesla V100 PCIe 16 GB GPU.

The results are shown in Table S1. We note that the worst performing model, for both descriptors, is the MLP. We do believe that it would be possible to improve the neural network, e.g. by changing its architecture and by tweaking the optimization parameters even more carefully. Still, the training speed and good performance of Gaussian Process models were enough for us to decide to use a Gaussian Process model. The best performing models all yield a similar mean absolute error on the validation set. Between the two descriptors, we have opted to use the Coulomb Matrix as is widely known, fast to compute, and as general as the BADM descriptor. For the Coulomb Matrix, both the Matern(5/2) and the Squared Exponential kernel perform equally well, but training with the latter often yielded unstable results. As the performance of GP with Matern(5/2) kernel and CM descriptor is within the best performing models, we have used this combination of featurization and model to build our regression models for the vacuum excitation energy of chlorophylls.

Conformational Freedom of Chlorophylls *a* and *b*

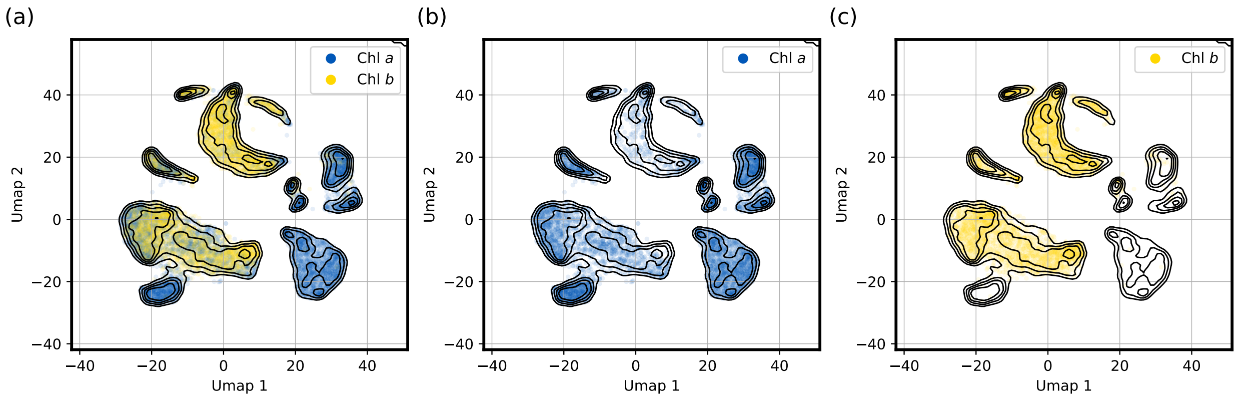

Figure S1: UMAP projection of the Coulomb Matrices of Chls *a* and *b* in LHCII. (a) Projection of Chl *a* and *b*. (b) Projection of only Chl *a*. (c) Projection of only Chl *b*.

The conformational freedom of Chl *a* and Chl *b* can be visualized by obtaining their Coulomb Matrices all together, and projecting them in a low-dimensional manifold. In order to perform this kind of analysis, Coulomb Matrices must have the same dimensionality (i.e., they have to be computed on the same number of atoms). As such, we have computed the Coulomb Matrices by ignoring the hydrogen atoms, in the same way we compute the matrices for the vacuum ML model, and by additionally removing the aldehydic group present in Chl *b*. The low-dimensional manifold was determined with the UMAP algorithm, and performed in practice with the `umap-learn` Python package.[S6] The UMAP projection was computed considering 15 nearest neighbors, using a minimum distance of 0.5 and a spread of 10, all other parameters left as their default value as provided by `umap-learn`.

The low-dimensional projection is visualized in Figure S1, where points belonging to Chl *a* and Chl *b* are represented in blue and yellow, respectively. The larger conformational freedom of Chl *a* is evident from the projection.

Scan Over an Improper Dihedral of a Chlorophyll *a*

In order to test the vacuum ML model, we have performed a scan over an improper dihedral of a Chl *a*. The scan is performed over the atoms NA, C1, MG, and C4. Scanning over this improper dihedral corresponds to shifting the position of the NA atom from one side to the other of the Chl plane. The scan is performed on a Chl *a* in vacuum. At each step of the scan, a MM optimization is carried out that relaxes the other internal degrees of freedom. The excitation energy is computed along the scan with TD-DFT M062X/6-31G(d). The Chl tail is excluded from the excited state calculation.

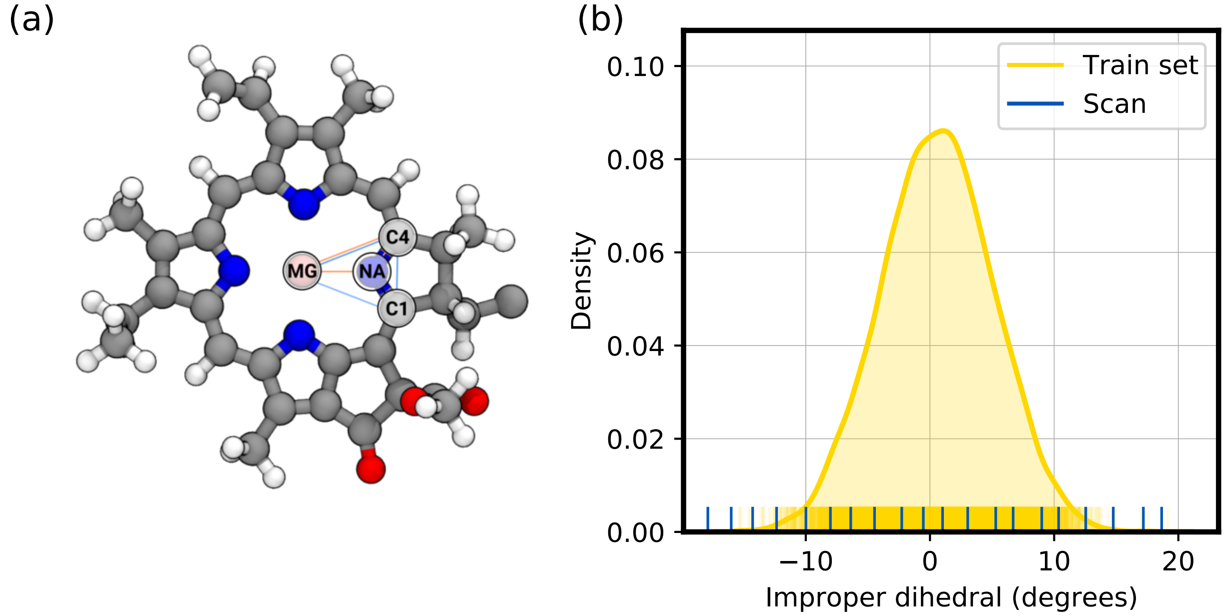

Figure S2: Scan over an improper dihedral of a Chlorophyll *a*. (a) Chlorophyll atoms used to define the improper dihedral. Colored lines connecting the atoms identify the the two planes, the angle of which is computed. (b)Comparison of the improper dihedral values in the train set and along the improper dihedral scan. Train set value are summarized with a kernel density estimation (KDE), represented in yellow. A shaded yellow bar is reported for each value in the training set on the bottom of the plot. Blue bars are reported for the improper dihedral values along the scan.

Ignoring Internal Coordinates in the Electrostatic Embedding ML Model

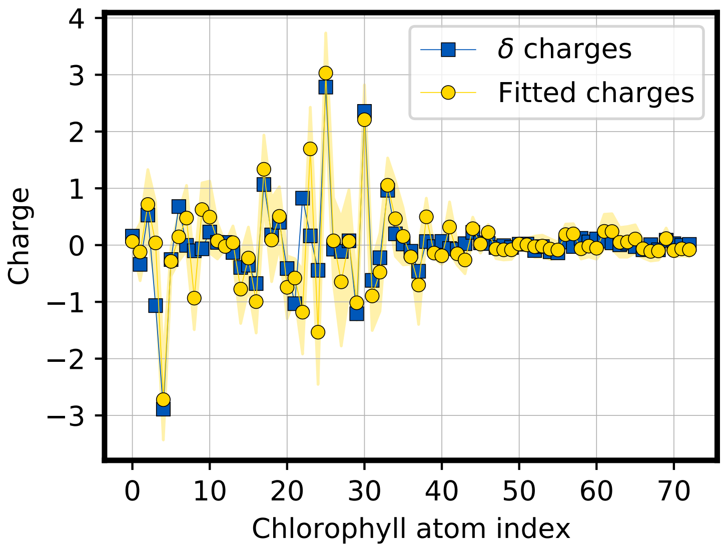

Figure S3: Comparison of δ charges (i.e., the charges that best fit the difference between excited and ground state densities), reported as blue squares, and the charges fitted by the linear EE ML model trained on MM electrostatic potentials only, represented in yellow circles. Charges are compared for Chl *a*. The yellow shaded region represents the uncertainty interval, computed for the i-th charge as $1.96 \times \mathrm{Std_B}[q_i]$, where $\mathrm{Std_B}[\cdot]$ represents the standard deviation computed from 100 bootstrap fits.

As explained in the main text, incorporating internal degrees of freedom in the EE ML model is essential in order to fit the correct response of the QM subsystem to the environment's electrostatics. The reason is that, when in different geometries (e.g., in a planar or a distorted conformation), the electron density of the chromophore will respond differently to the action of an external potential.

The importance of including internal coordinates can be appreciated by removing them from the EE ML model, obtaining a new model that is trained only on the MM electrostatic potential to predict the electrochromic shift. As detailed in the text, the kernel that acts on MM potentials is a linear kernel. If the internal coordinates are ignored, then, the Gaussian Process model becomes equivalent to a linear model. The coefficients learned from the linear

model can be thought of as effective atomic charges, interacting with the MM electrostatic potential to yield the electrochromic shift. The analogy is evident if one plots the learned coefficients, as in Figure S3 (yellow points). Figure S3 also plots the δ charges (blue squares), obtained as the difference between the charges that best fit the excited state density and the charges that best fit the ground state density. δ charges are computed with TD-DFT M062X/6-31G(d), and are used in the Charge Density Coupling (CDC)[S7] method to estimate the electrochromic shift through their interaction with the MM potential.

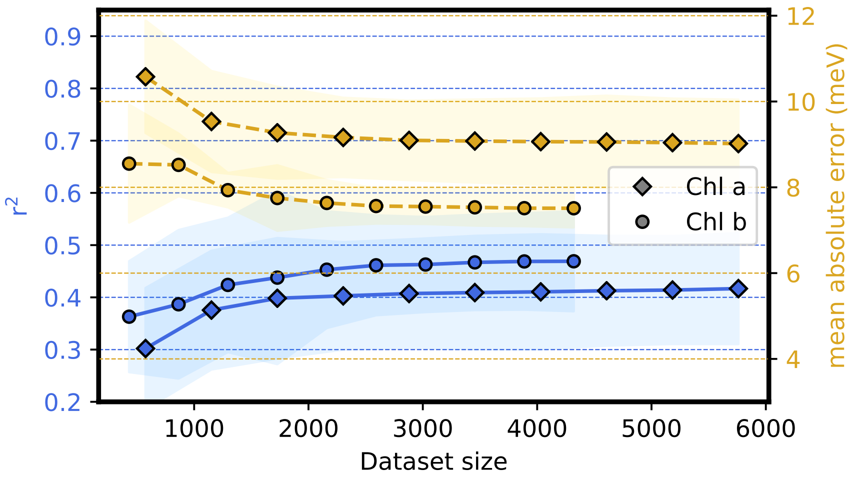

Figure S4: Learning curves for the electrochromic shift $\hat{\epsilon}_{\text{shift}}$ of chlorophylls in LHCII, as estimated by a linear EE ML model trained on MM electrostatic potentials only. Blue lines report the Pearson's r squared, yellow lines report the mean absolute error (MAE), both evaluated on the validation test with five-fold cross-validation (CV-5). The uncertainty is computed as twice the standard deviation of the validation score, and shown as a shaded region around the corresponding curve. The horizontal axis reports the dataset size used to perform CV-5. Diamond markers correspond to Chl a, while circles correspond to Chl b.

If the linear EE ML model is employed to predict the electrochromic shift, the performance is far worse than what is obtained with the full EE ML model. This is shown in Figure S4, where both the MAE and the Pearson's r^2 illustrate the insufficiency of the model (the MAE does not get below 7 meV, while in the EE ML model reaches 4 meV, and the r^2 is at most ~ 0.45, while in the EE ML model reaches ~ 0.9). Learning curves are computed with `scikit-learn`, performing a five-fold cross-validation (CV-5) for each dataset size, and employing a ridge-regularized linear model that selects the regularization strength

with efficient leave-one-out cross-validation (LOO-CV). The intercept is not fitted by the model.

Molecular Dynamics of a Chlorophyll *a* in Methanol

A MD simulation of a Chl *a* in methanol has been performed in order to thoroughly sample the pigment and solvent degrees of freedom. This simulation has been run in order to test the EE ML model on out-of-sample geometries. Simulations details are provided below.

The Chl *a* starting structure has been extracted from the LHCII MD simulation employed to construct the training dataset. The chlorophyll has been solvated in an octahedral box of methanol molecules, extending up to 25 Å from the pigment, for a total of 4344 methanol molecules. Parameters from Chl *a* were taken from the literature,[S8] and we have used methanol parameters from Ref S9 as available in AMBER 18.[S10] The system has been minimzed and heated previous to running the production run. Solvent minimization was carried out in 10000 steps, the first 5000 of which were performed with steepest descent, while the latter 5000 with conjugate gradient, keeping the Chl *a* fixed with harmonic restraints with a force constant of 10 kcal mol^{-1} $Å^{-1}$. Minimization has been carried out in the NVT ensemble. After the minimization step, the system was heated from 0 K to 300 K in a 100 ps long NPT simulation. Finally, the production run has been extended for 1 μs in the NVT ensemble, with an integration time step of 2 fs, employing SHAKE to place holonomic constraints on hydrogen atoms. The Langevin thermostat has been employed to keep the temperature fixed. Long range electrostatics was computed with the Particle Mesh Ewald (PME). All simulations were run with the AMBER 18 suite.[S10]

Site Energies and Couplings in CP29-WT and CP29-H111N

Site energies and couplings in CP29-WT and its mutant CP29-H111N can be estimated rapidly and reliably with our ML model. We have computed the site energy of every chlorophyll in both the WT and the mutant with our polarizable ML model. Electronic couplings have been computed with our regularized linear ML model presented in Ref.[S11] As shown in Refs.,[S11,S12] vacuum couplings are a good approximation to the polarizable ones by virtue of a cancellation of effects, in the particular case of Chls embedded in LHCs. For this reason and to further speed up the calculations, couplings reported herein are vacuum ones. Site energies and couplings have been computed on a classical MD trajectory of CP29-WT and CP29-H111N embedded in a membrane, already employed in previous works,[S13–S15] on 3000 frames for both CP29-WT and CP29-H111N (6000 frames in total). We also note that, as our linear model[S11] predicts TrEsp charges, as a by product we also obtain the transition dipoles associated with the transition as $\mu^{\mathrm{tr}} = \sum_i q_i^{\mathrm{tr}} \mathbf{r}_i$, where q_i^{tr} is the TrEsp charge of the i-th atom, and $\mathbf{r}_i$ its cartesian coordinates. Couplings have been computed between chlorophyll pairs within 20 Å. The environment was included up to 30 Å from each chlorophyll, and was allowed to polarize up to 15 Å. Site energies and couplings have been further block-averaged on a window of 10 frames, in order to average the fast fluctuations due to fast internal motions of the pigments. The distributions of the site energies and couplings are reported in Figure S5.

Calculation of the Absorption Spectrum of CP29-WT and CP29-H111N

The absorption spectrum has been calculated with the Full Cumulant Expansion (FCE) formalism, using the Fortran 90 implementation[S17] presented in Ref. S18. Details on the

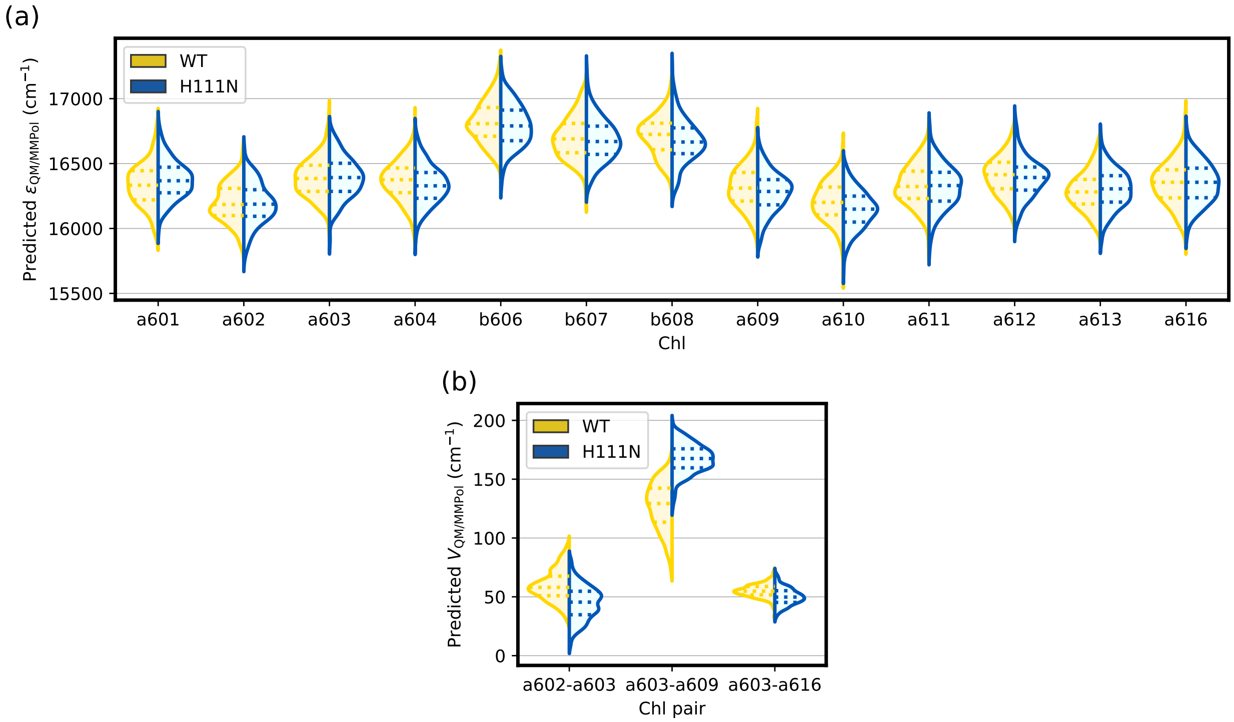

Figure S5: Site energies and electronic couplings in CP29-WT (yellow) and CP29-H111N (blue). (a) Violin plot showing the distribution of the predicted site energies of chlorophylls in CP29-WT and CP29-H111N. The site energy values are computed with our polarizable ML model as explained in the main text. (b) Violin plot showing the distribution of the electronic couplings of Chl a603 with nearby chlorophylls. Only the biggest couplings are shown. The electronic coupling is computed in the TrEsp approximation,[S16] employing TrEsp charges predicted with our ML model presented in Ref.[S11] In each violin plot, horizontal dotted lines indicate the quartiles of the distribution.

FCE for the calculation of optical spectra can be found in Refs. S18,S19.

In our work, the electronic Hamiltonian of the aggregate:

$$\mathcal{H} = \sum_i \epsilon_i |i\rangle\langle i| + \sum_{ij} V_{ij} |i\rangle\langle j| \tag{1}$$

has been assembled by estimating site energies ϵ_i and couplings V_{ij} with the ML models presented in the main text (for the site energies) and in Ref. S11. Site energies have been computed with our polarizable ML model, and couplings have been computed with our vacuum ML model as explained in Section . Carotenoids were not included in the calculation of the absorption spectrum.

The absorption spectrum in the FCE formalism is computed as:

$$A(\omega) = \omega \Re \int_0^\infty dt e^{i\omega t} \sum_{ij} \mathbf{M}_{ij} \mathbf{I}_{ij}(t) \tag{2}$$

where $\mathbf{I}(t)$ is the absorption tensor of the aggregate, and $\mathbf{M}$ is the dipole strength matrix. Transition dipoles have been computed with our linear ML model[S11] predicting TrEsp charges as $\mu_i = \sum_{a \in i} q_a^{\text{tr}} \mathbf{r}_a$.

The absorption tensor $\mathbf{I}(t) = e^{-iH_S t} e^{-\mathbf{K}(t)}$ is expressed in terms of the FCE lineshape matrix $\mathbf{K}(t)$, defined as:

$$K_{\mu\nu} = \sum_\alpha \sum_n X_n^{\mu\alpha} X_n^{\alpha\nu} \int_0^t dt_2 \int_0^{t_2} dt_1 e^{i\omega_{\mu\alpha} t_2 - i\omega_{\nu\alpha} t_1} C_n(t_2 - t_1) \tag{3}$$

where μ, ν, and α indicate the exciton state, $X_n^{\mu\alpha} = c_n^{\mu *} c_n^\alpha$ is the product of the exciton coefficients c_n^μ, obtained by diagonalizing the exciton Hamiltonian Eq. (1), and $C_n(t)$ is the autocorrelation function of the energy gap for the n-th pigment:

$$C_n(t) = \frac{1}{\pi} \int_0^\infty d\omega \left[\coth\left(\frac{\beta\hbar\omega}{2}\right) \cos(\omega t) - i \sin(\omega t) \right] \tilde{C}_n(\omega) \tag{4}$$

where $\tilde{C}_n(\omega)$ is the spectral density of the n-th pigment. In this work, we have used a

single spectral density for both Chl a and Chl b, derived through the vertical gradient (VG) approach for some frames of a Chl a in CP29. The spectral density is computed as:

$$\tilde{C}_n(\omega) = \sum_{\xi} \frac{1}{\hbar\omega_\xi} \left(\frac{\partial H}{\partial q_\xi}\right)^2 \frac{\omega_\xi^2 \omega \gamma_\xi}{\left(\omega^2 - \omega_\xi^2\right)^2 + \omega^2 \gamma_\xi^2} \tag{5}$$

where q_ξ are normal modes, the gradient $\partial H/\partial q_\xi$ is taken with respect to the excited state potential energy surface, and γ_ξ are damping factors set to 5 cm^{-1}. The normal mode frequencies and Huang-Rhys factors obtained with the VG approach are provided in a Zenodo repository.[S20] In addition, we have modeled the low-frequency part of the spectral density with an overdamped Brownian term with reorganization energy of 30 cm^{-1} and a damping constant of 5 cm^{-1}.

The final absorption spectrum is then computed as an average over multiple homogeneous spectra, each computed for different MD frames by estimating the excitonic parameters with our ML models. Additionally, for each frame included in the calculation, five realization of the static disorder are obtained by shifting the site energies by a shift $s \sim \mathcal{N}(0, \sigma_{dis})$, with $\sigma_{dis} = 40.0$ cm^{-1}.

$$\tilde{A}(\omega) = \sum_{f}^{N_f} \sum_{d}^{N_d} A_{f,d}(\omega) \tag{6}$$

where the sum runs over all the MD frames for which an homogeneous spectrum $A_{f,d}(\omega)$ for frame f and static disorder realization d has been calculated. For both CP29-WT and CP29-H111N, $N_f = 300$ and $N_d = 5$.

References

(S1) Rupp, M.; Tkatchenko, A.; Müller, K.-R.; von Lilienfeld, O. A. Fast and Accurate Modeling of Molecular Atomization Energies with Machine Learning. *Phys. Rev. Lett.* **2012**, *108*, 058301.

(S2) Huang, B.; von Lilienfeld, O. A. Communication: Understanding molecular representations in machine learning: The role of uniqueness and target similarity. *J. Chem. Phys.* **2016**, *145*, 161102.

(S3) Matthews, A. G. d. G.; van der Wilk, M.; Nickson, T.; Fujii, K.; Boukouvalas, A.; León-Villagrá, P.; Ghahramani, Z.; Hensman, J. GPflow: A Gaussian process library using TensorFlow. *J. Mach. Learn. Res.* **2017**, *18*, 1–6.

(S4) Häse, F.; Valleau, S.; Pyzer-Knapp, E.; Aspuru-Guzik, A. Machine learning exciton dynamics. *Chem. Sci.* **2016**, *7*, 5139–5147.

(S5) Paszke, A.; Gross, S.; Chintala, S.; Chanan, G.; Yang, E.; DeVito, Z.; Lin, Z.; Desmaison, A.; Antiga, L.; Lerer, A. Automatic differentiation in PyTorch. NIPS 2017 Workshop Autodif. 2017.

(S6) McInnes, L.; Healy, J.; Melville, J. UMAP: Uniform Manifold Approximation and Projection for Dimension Reduction. 2020; arXiv:1802.03426. arXiv.org ePrint archive. https://arxiv.org/abs/1802.03426 (accessed Jan 2, 2023).

(S7) Adolphs, J.; Müh, F.; Madjet, M. E.-A.; Renger, T. Calculation of pigment transition energies in the FMO protein: From simplicity to complexity and back. *Photosynth Res* **2008**, *95*, 197–209.

(S8) Zhang, L.; Silva, D.-A.; Yan, Y.; Huang, X. Force field development for cofactors in the photosystem II. *J. Comput. Chem.* **2012**, *33*, 1969–1980.

(S9) Cieplak, P.; Caldwell, J.; Kollman, P. Molecular mechanical models for organic and biological systems going beyond the atom centered two body additive approximation: aqueous solution free energies of methanol and N-methyl acetamide, nucleic acid base, and amide hydrogen bonding and chloroform/water partition coefficients of the nucleic acid bases. *J. Comput. Chem.* **2001**, *22*, 1048–1057.

(S10) Case, D. A.; Ben-Shalom, I. Y.; Brozell, S. R.; Cerutti, D. S.; Cheatham, T. E.; III,; Cruzeiro, V. W. D.; Darden, T. A.; Duke, R.; Ghoreishi, D.; Gilson, M. K.; Gohlke, H.; Goetz, A. W.; Greene, D.; Harris, R.; Homeyer, N.; Izadi, S.; Kovalenko, A.; Kurtzman, T.; Lee, T. S.; LeGrand, S.; Li, P.; Lin, C.; Liu, J.; Luchko, T.; Luo, R.; Mermelstein, D. J.; Merz, K. M.; Miao, Y.; Monard, G.; Nguyen, C.; Nguyen, H.; Omelyan, I.; Onufriev, A.; Pan, F.; Qi, R.; Roe, D. R.; Roitberg, A.; Sagui, C.; Schott-Verdugo, S.; Shen, J.; Simmerling, C. L.; Smith, J.; Salomon-Ferrer, R.; Swails, J.; Walker, R. C.; Wang, J.; Wei, H.; Wolf, R. M.; Wu, X.; Xiao, L.; York, D. M.; Kollman, P. A. AMBER 18. 2018; University of California, San Francisco.

(S11) Cignoni, E.; Cupellini, L.; Mennucci, B. A fast method for electronic couplings in embedded multichromophoric systems. *J. Phys.: Condens. Matter* **2022**, *34*, 304004.

(S12) Friedl, C.; Fedorov, D. G.; Renger, T. Towards a quantitative description of excitonic couplings in photosynthetic pigment–protein complexes: quantum chemistry driven multiscale approaches. *Phys. Chem. Chem. Phys.* **2022**, 5014–5038.

(S13) Lapillo, M.; Cignoni, E.; Cupellini, L.; Mennucci, B. The energy transfer model of nonphotochemical quenching: Lessons from the minor CP29 antenna complex of plants. *Biochim. Biophys. Acta, Bioenerg.* **2020**, *1861*, 148282.

(S14) Cignoni, E.; Slama, V.; Cupellini, L.; Mennucci, B. The atomistic modeling of light-harvesting complexes from the physical models to the computational protocol. *J. Chem. Phys.* **2022**, *156*, 120901.

(S15) Guarnetti Prandi, I.; Sláma, V.; Pecorilla, C.; Cupellini, L.; Mennucci, B. Structure of the stress-related LHCSR1 complex determined by an integrated computational strategy. *Commun Biol* **2022**, *5*, 145.

(S16) Madjet, M. E.; Abdurahman, A.; Renger, T. Intermolecular Coulomb Couplings from Ab Initio Electrostatic Potentials: Application to Optical Transitions of Strongly

Coupled Pigments in Photosynthetic Antennae and Reaction Centers. *J. Phys. Chem. B* **2006**, *110*, 17268–17281.

(S17) Cupellini, L.; Lipparini, F. FCE Program to compute optical spectra with the Full Cumulant Expansion. 2020; `https://doi.org/10.5281/zenodo.3900200`.

(S18) Cupellini, L.; Lipparini, F.; Cao, J. Absorption and Circular Dichroism Spectra of Molecular Aggregates With the Full Cumulant Expansion. *J. Phys. Chem. B* **2020**, *124*, 8610–8617.

(S19) Ma, J.; Cao, J. Förster resonance energy transfer, absorption and emission spectra in multichromophoric systems. I. Full cumulant expansions and system-bath entanglement. *J. Chem. Phys.* **2015**, *142*, 094106.

(S20) Cignoni, E.; Cupellini, L.; Mennucci, B. excipy: Machine learning models for a fast estimation of excitonic Hamiltonians. 2023; DOI: 10.5281/zenodo.7503183.
